# High-yield production of “difficult-to-express” proteins in a continuous exchange cell-free system based on CHO cell lysates

**DOI:** 10.1038/s41598-017-12188-8

**Published:** 2017-09-15

**Authors:** Lena Thoring, Srujan K. Dondapati, Marlitt Stech, Doreen A. Wüstenhagen, Stefan Kubick

**Affiliations:** 1 Fraunhofer Institute for Cell Therapy and Immunology (IZI), Branch Bioanalytics and Bioprocesses (IZI-BB), Am Mühlenberg 13, D-14476 Potsdam, Germany; 2 Institute for Biotechnology, Technical University of Berlin (TUB), Gustav-Meyer-Allee 25, 13355 Berlin, Germany

## Abstract

Cell-free protein synthesis (CFPS) represents a promising technology for efficient protein production targeting especially so called “difficult-to-express” proteins whose synthesis is challenging in conventional *in vivo* protein production platforms. Chinese hamster ovary (CHO) cells are one of the most prominent and safety approved cell lines for industrial protein production. In this study we demonstrated the ability to produce high yields of various protein types including membrane proteins and single chain variable fragments (scFv) in a continuous exchange cell-free (CECF) system based on CHO cell lysate that contains endogenous microsomal structures. We showed significant improvement of protein yield compared to batch formatted reactions and proved biological activity of synthesized proteins using various analysis technologies. Optimized CECF reaction conditions led to membrane protein yields up to 980 µg/ml, which is the highest protein yield reached in a microsome containing eukaryotic cell-free system presented so far.

## Introduction

Manufacturing of recombinant proteins is a highly important area in today’s pharmaceutical industry for producing complex therapeutics. A broad range of products with high relevance include monoclonal antibodies, growth factors, hormones, blood factors and enzymes^1,2^. Various cell lines were adapted and applied to biotechnological processes, including bacteria, insect, yeast and mammalian cells as host organisms, to produce potential drugs^3^. Since more than 30 years cell fermentation based on chinese hamster ovary (CHO) cells is one of the most commonly used and well established system for biopharmaceutical manufacturing processes^4^. The proven ability of CHO cells to produce complex recombinant proteins and to perform human like posttranslational modifications underlines the advantages connected with these host cells^5^. Moreover, the systematic elucidation of the CHO cell genome provides new opportunities for future development and optimization of this platform^6^.

Establishing an *in vivo* protein production process usually requires a long development time and a high degree of resources. Special types of proteins including the class of membrane proteins and some antibody types are difficult to express in cell based systems. Especially membrane proteins which are involved in important metabolic processes constitute potential, relevant drug targets as their dysfunction due to mutations could be detected in various severe diseases^7^. During the last years cell-free protein synthesis (CFPS) has come to the point where difficulties aroused by *in vivo* production of recombinant proteins^8^. The conversion of cellular organisms to a cell lysate containing components necessary for protein translation enables a rapid protein production process with an open system character to allow for adjustment of reaction conditions required for each individual protein^9,10^. Various cell-free protein synthesis systems are currently available differing in the origin of the cell lysate and the reaction mode. As a result, novel systems harboring different reaction lifetimes, obtained protein yields and last but not least cost efficiencies have emerged. Regardless of lysate origin, CFPS reactions could be conducted in batch and continuous exchange mode, whereby the batch mode represents a simple and fast one pot reaction^11^. Continuous exchange cell-free systems (CECF) are based on a reaction and a feeding chamber separated by a semipermeable membrane and thereby provide the advantage of energy delivery and removal of inhibitory byproducts to the prolong reaction time in order to increase total protein yields^12^. Recent studies demonstrate protein synthesis in numerous CECF systems exemplarily based on *E*. *coli* lysates^13^, wheat germ lysates^14^, tobacco lysates^15^ and insect cell lysates^16^. Each system show specific advantages and disadvantages. Deploying *E*. *coli* based cell-free systems enable high protein yields due to the efficient translation machinery, while nanodiscs, liposomes or detergents are usually supplemented to the reaction for the production of proper folded and functional membrane proteins^10,13,17^. Numerous reports are available showing the applications of *E*.*coli* batch and CECF systems^10,18–21^ and demonstrating the possibility to use the produced proteins for structural estimation^13,22–24^. This underlines the relevance of *E*.*coli* cell-free system in combination with exemplarily nanodiscs for the production of non-modified membrane proteins, which possesses a cost efficient and fast protein production system. Eukaryotic cell-free systems for the production of posttranslationally modified proteins are currently available and dedicated eukaryotic systems harbor endogenous microsomal structures derived from the endoplasmatic reticulum^25,26^. Regarding to the synthesis of membrane proteins and posttranslationally modified proteins, a direct integration into a nature like lipid milieu is possible using such eukaryotic microsomal systems, which contain various ER based enzymes essential for posttranslational modifications and protein folding^27^. Some examples for microsome-containing cell-free expression platforms are based on Tobacco BY-2 extracts^28^, Yeast extract^29^, Spodoptera frugiperda extracts^25^, extracts from cultured CHO cells^26^ and extracts from cultured human cell lines^30^. So far, these systems were mostly performed in the batch reaction mode resulting in protein yields up to 50 µg/ml^31^. An increase in protein yield, that is essential for further industrial applications and functionality assessments, was achieved applying the CECF format to *Sf*21 based CFPS whereby protein yields up to 285 µg/ml of the epidermal growth factor receptor and up to 700 µg/ml for virus envelope protein gp67 have been achieved^16,32^.

Until today CHO cell-free systems containing microsomal fractions were not performed in the CECF mode^26^. According to the high relevance of CHO cells for biopharmaceutical applications, we demonstrate the CHO CFPS in the CECF format for high yield protein production. In this study, we evaluate the potential to produce high yields of different classes of model proteins, including membrane proteins and single chain antibody fragments in a CHO CECF system. In this context, we examined the biological activity of cell-free synthesized proteins using different functionality assays.

## Results

### Development of a continuous exchange cell-free system based on CHO cell lysates

**Figure 1:**
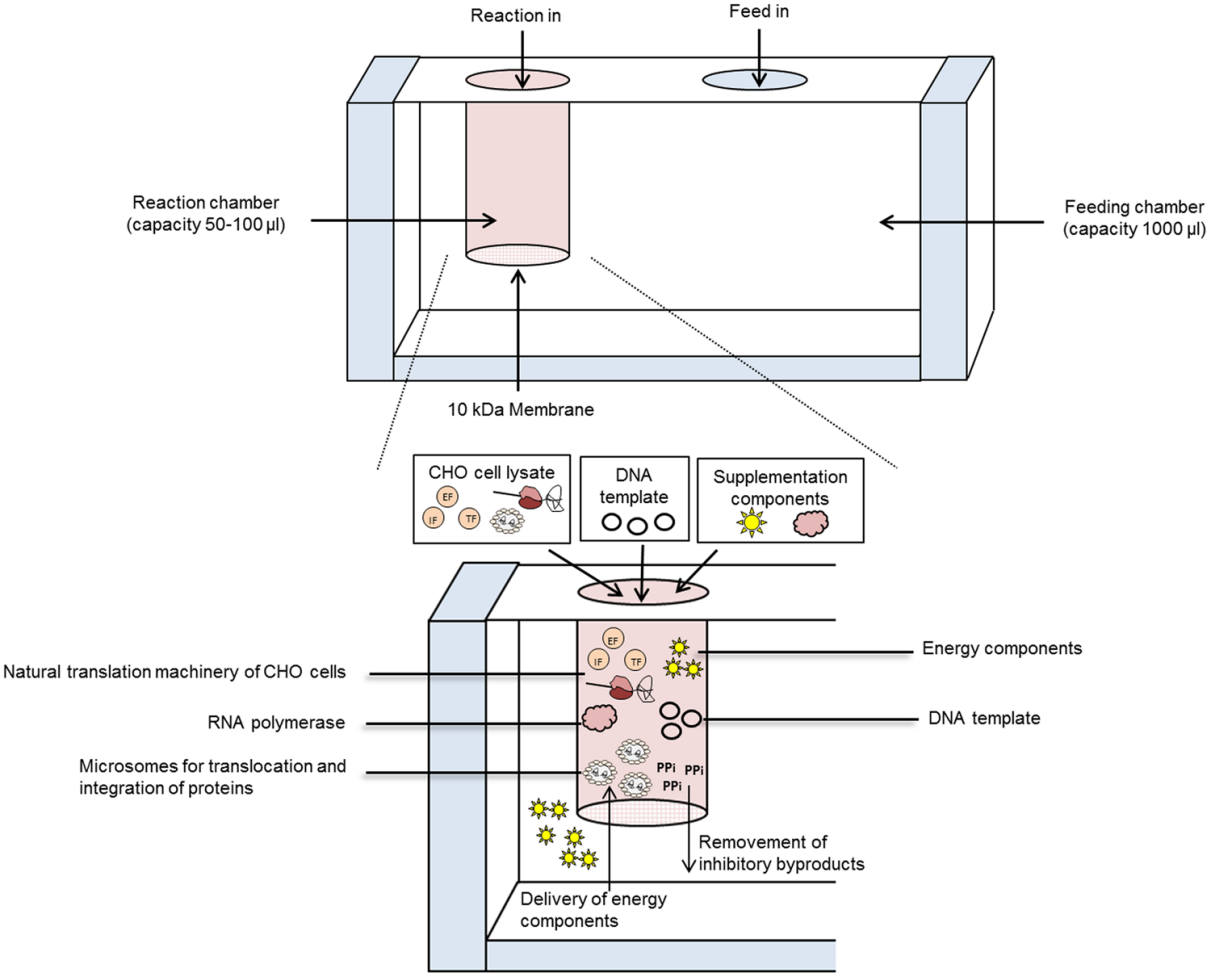
Schematic overview of continuous exchange cell-free (CECF) protein synthesis based on CHO cell lysates. A two chamber reaction device (RiNA GmbH) serves as a basis for CECF protein production (upper part of the figure). The reaction and a feeding chamber are separated by a 10 kDa cutoff, semipermeable membrane enabling a semi continuous delivery of energy to the reaction chamber and a removal of inhibitory byproducts.

Nowadays, various cell-free systems of different cellular origin are available. They enable the synthesis of proteins in a broad concentration range. In previous studies we have demonstrated the synthesis of various proteins in cell-free systems based on *Sf*21 and CHO lysates harboring endogenous microsomal structures^25,33^. Meanwhile *Sf*21 based cell-free systems are available in batch and CECF reaction mode^16,34,35^. Until now, CHO cell-free systems harboring microsomal structures were only performed in batch mode^26^. For the development of the CHO cell-free system in CECF reaction mode, we decided to add a Cricket paralysis virus (CRPV) internal ribosomal entry site (IRES) to the 5′UTR of our target gene sequences which was reported to be advantageous concerning protein yield^31^ by enabling translation factor independent translation initiation. A schematic overview of the principle of CHO CECF reaction is presented in Fig. 1. The system is based on a two chamber reaction device consisting of a reaction and a feeding compartment, which enables a prolonged reaction time and therefore an increase in the derived protein yield. In this study a small scale dialysis device was used consisting of a 50 µl reaction chamber and a 1000 µl feeding chamber. Detailed information about the proteins synthesized in this study is given in supplementary table 1.

**Figure 2:**
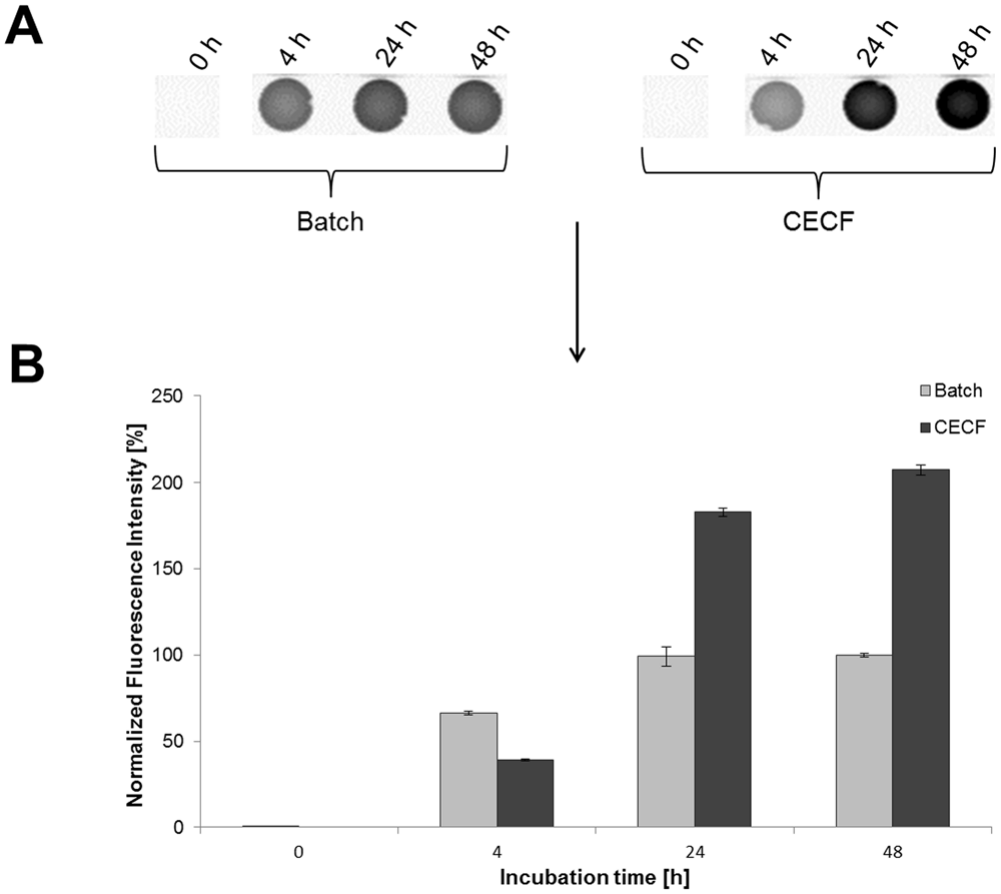
Time series analysis of batch and CECF reactions by quantification of produced fluorescent eYFP. The influence of reaction time (0 h, 4 h, 24 h, 48 h) on protein synthesis rate was analyzed in the batch and CECF reaction mode. Fluorescence was detected using a µ-Ibidi slide (**A**) and a Typhoon Trio + Variable Mode Imager (GE Healthcare) (Excitation 488 nm, emission filter 526 nm short-pass). The original image of the µ-Ibidi slide can be found in supplementary figure 7. (**B**) Quantification of fluorescence intensity was performed using Image Quant TL software (GE Healthcare). Fluorescence intensity was normalized with regard to the maximum batch value. Error bars represent the standard deviation of triplicate analysis.

A first comparison of the batch and CECF synthesis based on the CHO lysate system was performed using eYFP as a reporter protein. Incubation of the CECF reaction for 24 h and 48 h led to an increased fluorescence signal compared to batch reaction (Fig. 2A). After 4 h of reaction the fluorescence intensity of the batch sample was slightly higher than CECF. Nevertheless, increased fluorescence intensities after 24 h and 48 h were only detected for the CECF sample. Quantification analysis of received fluorescence signals demonstrated a significant increase (1.9-2.1-fold) of fluorescence intensity for CECF after 24 h and 48 h compared to batch reactions (Fig. 2B). Based on these results the incubation time of CECF reactions was set to 48 h.

### High yield production of functional Epidermal Growth Factor Receptor (EGFR)

**Figure 3:**
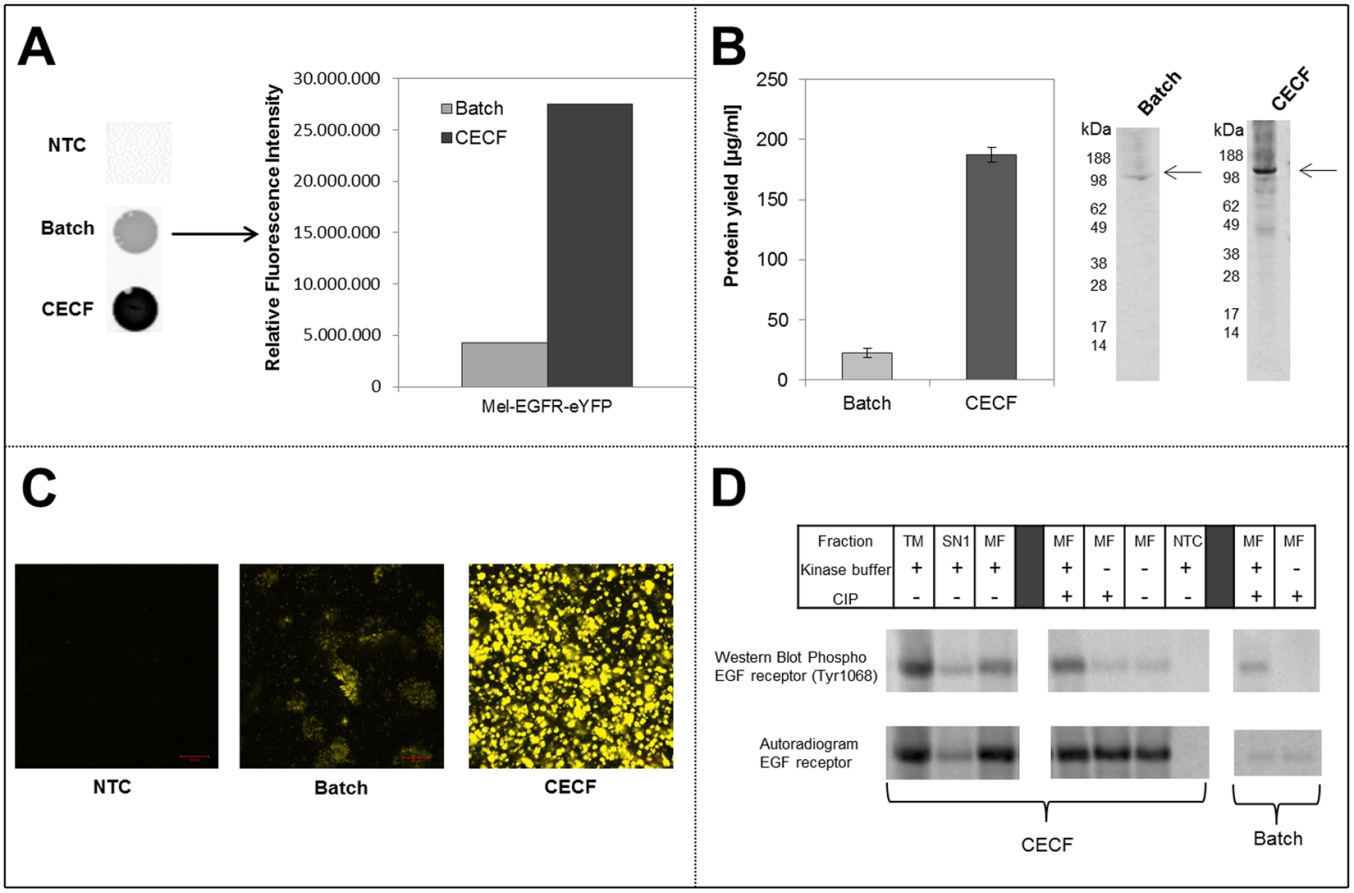
Comparison of Mel-EGFR-eYFP cell-free synthesis in the batch and the CECF reaction format. (**A**) Fluorescence analysis of Mel-EGFR-eYFP using a Typhoon Trio + Variable Mode Imager (GE Healthcare) (excitation 488 nm, emission filter 526 nm short-pass). Quantification of fluorescence intensity was performed using Image Quant TL software (GE Healthcare). Fluorescence intensity was normalized with regard to the batch value. No template control (NTC) contained CHO cell-free translation mixture without addition of DNA template. (**B**) Quantification of total protein yields by TCA precipitation of synthesized Mel-EGFR-eYFP and scintillation measurement. Error bars represent the standard deviation of triplicate analysis. Autoradiography of corresponding samples after electrophoretic separation. (**C**) Analysis of Mel-EGFR-eYFP intensity and localization in cell-free reactions using confocal laser scanning microscopy (CLSM). (Scale bar 10 µM) (**D**) Analysis of *in vitro* phosphorylation of Mel-EGFR-eYFP tyrosine 1068 residue by immunoblotting. Comparison of western blot, displaying the phosphorylated EGFR, and corresponding autoradiography detecting the total amount of EGFR. CECF samples were separated into translation mixture (TM), supernatant (SN1) and microsomal fraction (MF). To verify the specificity of the phosphorylation by EGFR kinase, samples were treated with calf intestinal phosphatase (CIP) to enable dephosphorylation of EGFR prior kinase buffer treatment. No template control (NTC) contained CHO cell-free translation mixture without supplementation of DNA template. Image analysis of immunoblot and quantitative comparison with the corresponding autoradiography of the blot is illustrated in supplementary Figure 1. The original image of the western blot and the corresponding autoradiogram can be found in supplementary figure 8.

The class of membrane proteins is of highest relevance for pharmaceutical research. Due to their complex structure membrane proteins belong to the class of so called difficult-to-express proteins which tend to aggregate during *in vivo* protein expression. Eukaryotic cell-free protein synthesis systems harboring endogenous microsomes offer an alternative option for membrane protein synthesis and direct integration into nature like membrane structures. We previously described the production of Epidermal Growth Factor Receptor (EGFR) in a microsome containing cell-free system based on *Sf*21 lysates^16^. In order to develop the CHO CECF system further we have evaluated and optimized the synthesis rate of EGFR and provided results with regards to functional assessment of EGFR autophosphorylation activity. Therefore, eYFP was fused to the C-terminal part of EGFR to enable a simple and fast analysis of protein using fluorescence imaging methods. Fluorescence imaging results revealed major intensity differences between batch and CECF reactions (Fig. 3A). The quantification of fluorescence signals using Image Quant TL software emphasized the visual outcome and demonstrated a 5.5 fold increase of fluorescence signal for CECF compared to batch reaction. Supplementation of reactions with ^14^C leucine allowed the quantification of EGFR protein yield. The yield determined in batch and CECF corresponded to the fluorescence analysis. An EGFR protein yield in the range of 187 ± 7 µg/ml was obtained representing an 8 fold increase compared to batch reaction (22 ± 4 µg/ml) (Fig. 3B). Autoradiography confirmed a protein band showing the expected molecular weight. Intensity differences between batch and CECF reaction were detected. To analyze the localization of cell-free synthesized EGFR, confocal laser scanning microscopy (CLSM) was applied to translation mixtures of batch and CECF reactions (Fig. 3C). A direct comparison of EGFR synthesized in batch and CECF reaction revealed an increased fluorescence signal in the CECF sample. Small, tightly packed microsomal structures were observed in the CECF sample whereas the fluorescence signal in batch reaction was widely distributed in the sample (Fig. 3C). Both samples were analyzed using the same microscopic setting, whereas this setting is almost below the threshold for the detection of batch derived fluorescent proteins. As a result, this led to the impression of widely distributed protein in the batch reaction. In contrast, EGFR-eYFP synthesized in the CECF format showed a dense labeling of the microsomes due to the high protein yield. This CLSM analysis gave a hind for the translocation of EGFR-eYFP into the microsomes.

During activation of EGFR, the receptors intrinsic tyrosine kinase activity is activated and results in phosphorylation of serval tyrosine residues, including tyrosine residue 1068. This enzymatic process performed by the intrinsic kinase activity is also called autophosphorylation or self-phosphorylation. As a consequence, phosphorylated adapter proteins bind to EGFR and subsequently activate different signal transduction pathways^36^. In general, EGFR is activated by binding of a receptor specific ligand. Apart from the conventional activation it was demonstrated that a ligand independent mechanism exists, which seems to be promoted by high densities of available EGFR^37^. To assess the functionality of EGFR synthesized in the CHO CECF system, phosphorylation of tyrosine residue 1068 was exemplarily analyzed by a kinase assay followed by immuno blotting (Fig. 3D). Samples were treated with kinase buffer to enable phosphorylation of EGFR. Non treated samples were prepared to estimate background autophosphorylation activity. Phosphorylation bands were visible for all fractions of the CECF reaction, which were treated with kinase buffer, including translation mixture, supernatant and microsomal fraction. In the batch reaction the microsomal fraction showed significant phosphorylation bands. The intensity of phosphorylation bands differed between the analyzed fractions. Highest intensity was detected in the translation mixture of the CECF reaction, whereas a small reduction of intensity was observed in the microsomal fraction. Microsomal fraction of CECF samples displayed a higher signal intensity compared to the corresponding batch sample. Only low background phosphorylation was detected in the microsomal fraction which did not contain kinase buffer. The intensity of the phosphorylation band in the supernatant fraction of CECF was low compared to other fractions. The specificity of EGFR phosphorylation was verified by removing the phosphate moieties using calf intestinal phosphatase (CIP). As expected CIP treated samples showed only a reduced signal intensity of the phosphorylated protein band without addition of kinase buffer. Further supplementation of kinase buffer to the CIP treated sample led to an increase in the band intensity again. Corresponding autoradiography verified the presence of equal amounts of EGFR in the microsomal fractions of the CECF reactions. Quantification and comparison of phosphorylated and total EGFR was performed by image analysis of immunoblotting and autoradiography using Image Quant TL software as described (supplementary figure 1). Image analysis also revealed the increased amount of phosphorylated EGFR in the CECF reaction in comparison to corresponding batch samples (supplementary figure 1B).

**Figure 4:**
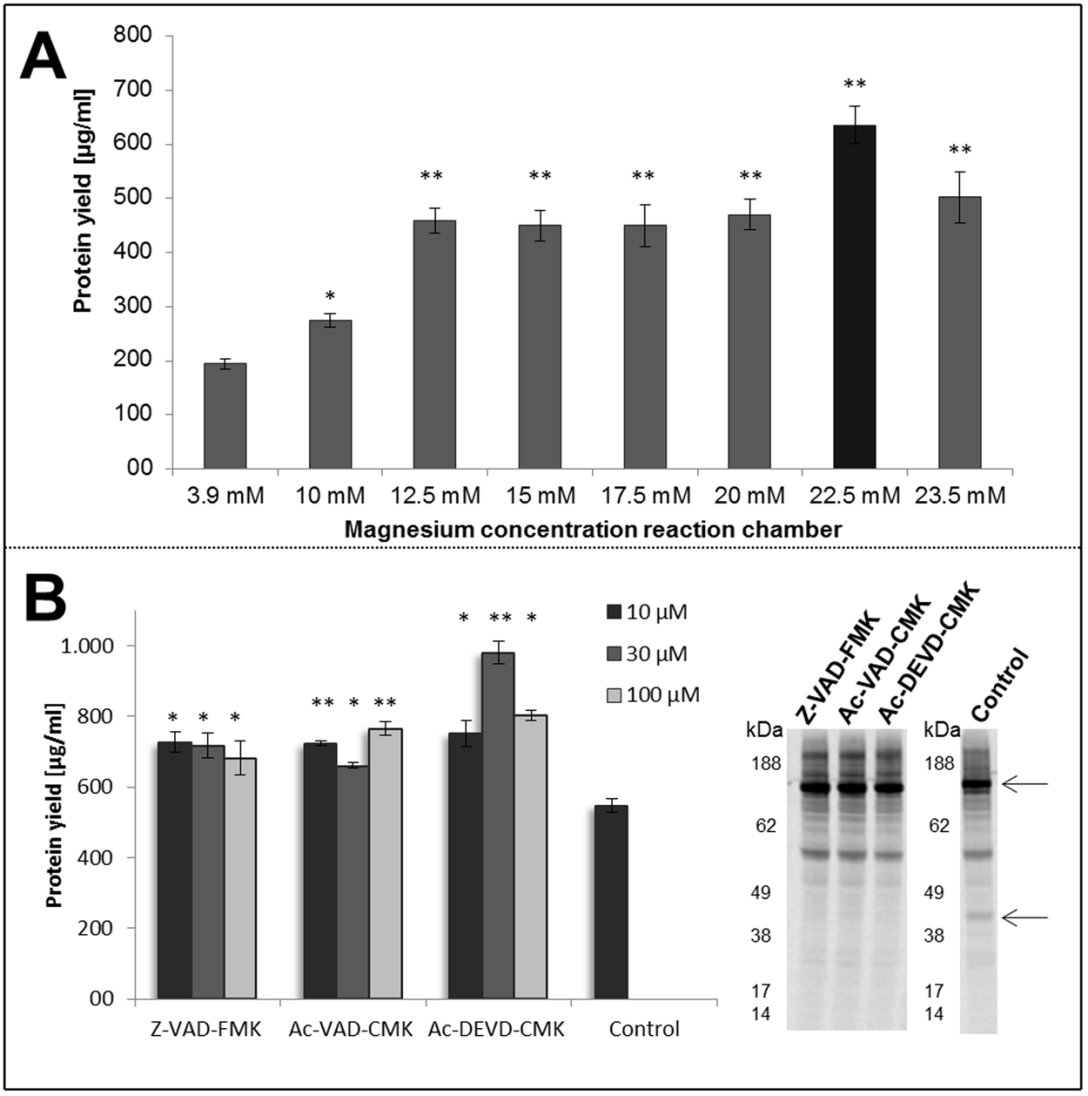
Optimization of Mel-EGFR-eYFP protein yields in the CECF reaction mode. (**A**) Influence of magnesium concentration in the reaction chamber during CECF reaction. Radio labeled Mel-EGFR-eYFP was used to determine protein yield. Error bars represent the standard deviation of triplicate analysis. Asterisks indicate the significance of deviation with regard to the control experiment (3.9 mM magnesium)(*p < 0.05; **p < 0.01). (**B**) Supplementation of caspase inhibitors Z-VAD-FMK, Ac-VAD-CMK, Ac-DEVD-CMK in different concentrations (10 µM, 30 µM, 100 µM) to CECF reaction. The original image of the autoradiogram can be found in supplementary figure 9. Preliminary experiments were performed to make a preselection for appropriate caspase inhibitors (supplementary figure 3). Total protein yields of EGFR-eYFP were determined. Autoradiography of corresponding samples after electrophoretic separation was demonstrated. Error bars represent the standard deviation of triplicate analysis. Asterisks indicate the significance of deviation with regard to the non-treated control experiment (*p < 0.05; **p < 0.01).

Our optimization process for CECF reactions included two strategies using EGFR as model protein. The first part was focused on optimization of magnesium concentration in the reaction chamber. In general, a final concentration of 3.9 mM magnesium (Mg^2+^) is applied to cell-free reactions based on CHO lysates and CRPV IRES constructs^31^. Transcription reaction in CECF coupled system is performed using T7 RNA polymerase. Crystal structure of this enzyme has provided evidence that two amino acid residues (Asp^537^ and Asp^812^) located near the catalytic center bind to metal ions to enable high catalytic activity of T7 RNA polymerase^38^. Therefore concentration dependency of CECF protein synthesis to Mg^2+^ was tested during the experimental optimization of CECF reaction (Fig. 4A). An adjustment of Mg^2+^ from 3.9 mM to 12.5 mM in the reaction chamber of the CECF system led to a 2.5 fold increase of protein yield of approximately 194 ± 10 µg/ml to 485 ± 35 µg/ml. An increase of Mg^2+^ concentration from 12.5 mM to 20 mM did not significantly enhance the protein yield while supplementation of 22.5 mM Mg^2+^ resulted in EGFR yields of around 635 ± 35 µg/ml, a 1.4 fold increase compared to the protein yield achieved with 20 mM Mg^2+^. In contrast, addition of Mg^2+^ beyond 23.5 mM resulted in a decrease of protein yield to the range of 501 ± 48 µg/ml. Based on these results, the following experiments were performed with 22.5 mM Mg^2+^ as optimized concentrations.

Next, we examined the influence of caspase inhibitors in the CHO CECF system. Caspases are cysteine proteases that cleave their substrates at aspartic residues^39^. We previously demonstrated the capability of specific caspase inhibitors to influence cell-free reactions positively^40^. In accordance to these early findings, we have supplemented CHO CECF reactions with selected caspase inhibitor types, which we have chosen after a comparative analysis of a multitude of different caspase inhibitors (supplementary figure 2). An increase in protein yield was detected for all chosen caspase inhibitors using various concentrations (10 µM, 30 µM, 100 µM) (Fig. 4). Supplementation of Ac-DEVD-CMK (30 µM) led to maximum raise in protein yield up to the range of 982 ± 30 µg/ml which is approximately 1.8 fold higher compared to the control without addition of caspase inhibitor. Autoradiography revealed a sharp EGFR product band with an apparent molecular weight of 160 kDa (Fig. 4 part B, upper arrow). Two faint extra bands were detected around 55 kDa and 40 kDa whereas the second protein band disappeared after supplementation of caspase inhibitors.

### Synthesis of single chain antibody variable fragment (scFv)

**Figure 5:**
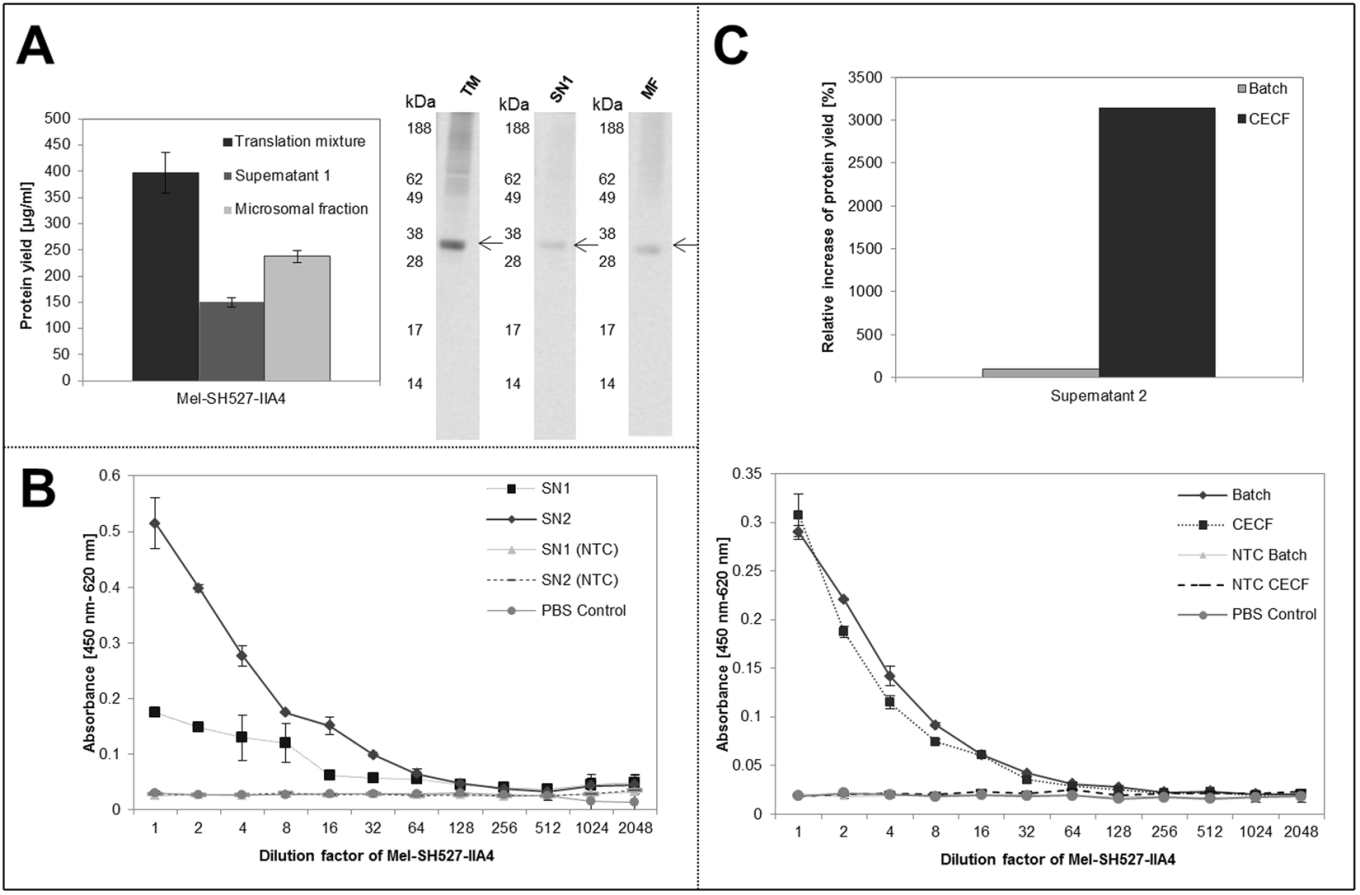
Production of single chain antibody variable fragment Mel-SH527-IIA4 in CECF based cell-free system. (**A**) Qualitative and quantitative analysis of translation mixture, supernatant 1 and microsomal fraction of produced Mel-SH527-IIA4. (**B**) Functional characterization by binding to corresponding antigene. Supernatant 1 (SN 1) and 2 (SN 2) fraction were compared regarding the activity of CECF synthesized Mel-SH527-IIA4. Supernatant 1 and 2 of no template control (NTC) and PBS control (PBS + 1%BSA) were analyzed to estimate background binding. SN1 fraction is derived by centrifugation of total translation mixture harboring non translocated proteins. SN2 fraction contained scFv translocated into the microsomes of CHO cell lysate, which were resolved by treatment with DDM. (**C**) Comparison of Mel-SH527-IIA4 (SN2 fraction) produced in batch and CECF format. Relative increase in protein yield of supernatant fractions 2 was calculated based on protein quantification. Error bars represent the standard deviation of triplicate analysis. An initial concentration of 0.5 ng/µl scFv was applied for each ELISA approach. A serial dilution of scFv was performed using 1% BSA in PBST.

Next, we investigated the possibility to synthesize antibody fragments (scFv) (Mel-SH527-IIA4) in the CHO based CECF format. Plasmids coding for SH527-IIA4 scFv contain a melittin signal sequence to enable efficient translocation of target proteins into microsomal vesicles. As has been already presented by Stech *et al*.,^40^, the presence of microsomes is necessary to obtain properly folded and functionally active scFvs in *Sf*21 based cell-free systems^41^. Determination of protein concentration revealed yields of Mel-SH527-IIA4 up to around 397 ± 30 µg/ml in the translation mixture, 150 ± 10 µg/ml in the supernatant fraction 1 and approximately 237 ± 10 µg/ml in the microsomal fraction (Fig. 5A). Corresponding protein bands were detected as distinct and homogeneous bands in the autoradiograph, showing the expected migration pattern. Functional activity of synthesized Mel-SH527-IIA4 was estimated by an antigen binding assay using an ELISA (Fig. 5B,C). A biotinylated peptide (SMAD2-P) was immobilized on a microtiter plate before application of cell-free synthesized Mel-SH527-IIA4 to the wells. No template controls (NTC) harboring fractions without DNA template and PBS control showed no significant signal. To underline the relevance of translocation of scFv into microsomal vesicles of CHO lysate, binding specificity of supernatant fraction 1 containing non-translocated Mel-SH527-IIA4 was analyzed in comparison to supernatant fraction 2, prepared by treatment of MF with 0.2% DDM, containing translocated and resolubilized Mel-SH527-IIA4. Supernatant fraction 2 showed a significantly higher binding specificity than supernatant fraction 1 (Fig. 5B), while a low activity of supernatant 1 fraction was detected. Using CECF synthesis, 31.4 fold increased scFv protein yields compared to batch reaction were obtained in supernatant fraction 2. Comparison of the activity of supernatant fraction 2 of scFv produced in batch and CECF reaction revealed equivalent binding capabilities using the same concentration of molecules (Fig. 5C). Therefore an around 31 fold higher amount of active scFv is produced in CECF reaction compared to batch format.

### Cell-free synthesis of KvAP

**Figure 6:**
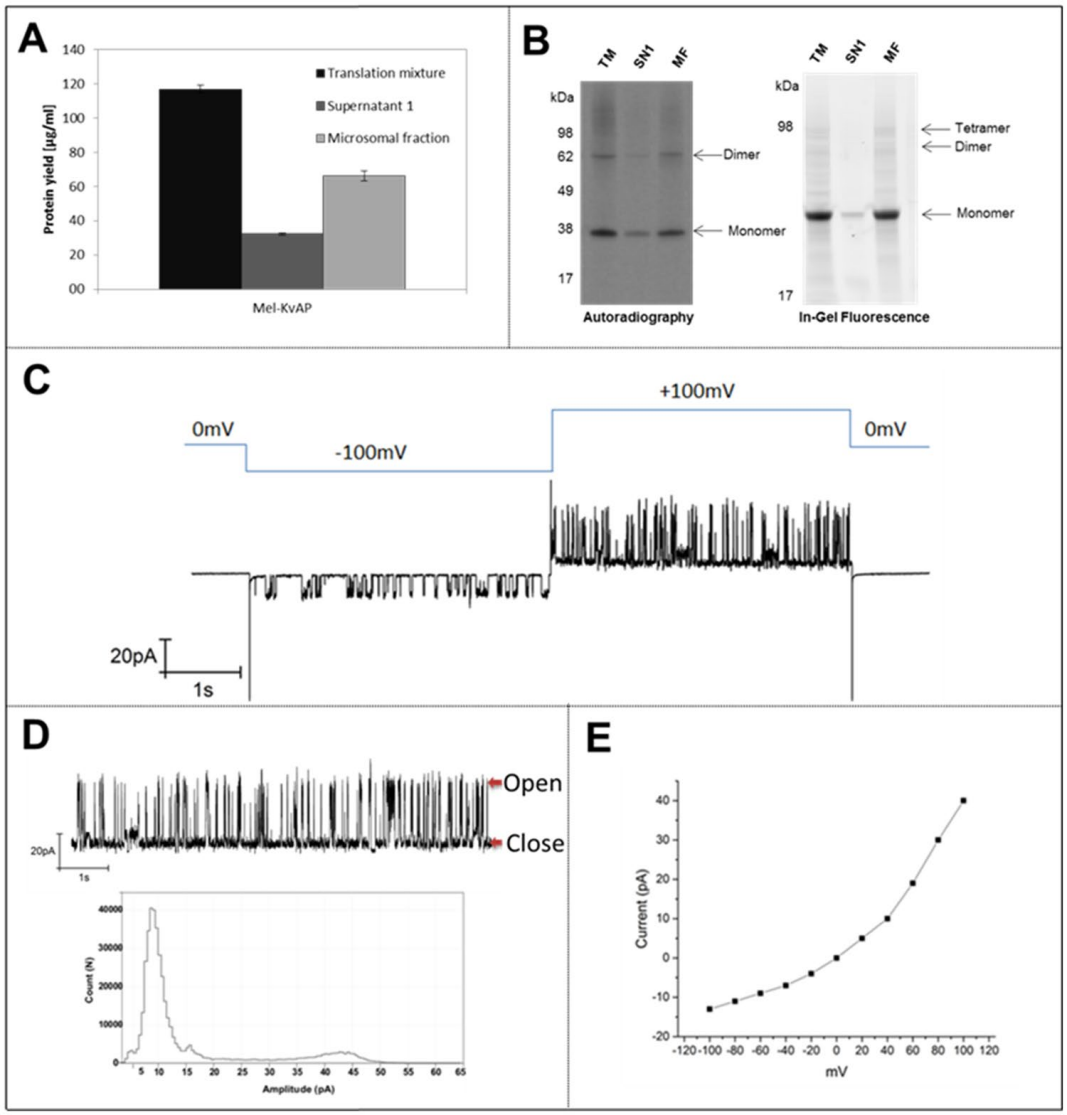
Synthesis of ion channel KvAP in CECF based reaction format. (**A**) Quantitative analysis of protein yield by scintillation measurements. Error bars represent the standard deviation of triplicate analysis. (**B**) Autoradiography and In-gel fluorescence of translation mixture, supernatant 1 and microsomal fraction of KvAP sample. Fluorescence samples were labeled by supplementation with BODIPY-TMR-Lys tRNA_Phe_ to reaction mixture and integration of lysine-dye-conjugate into protein sequence. Fluorescence analysis of labeled KvAP was accomplished by using a Typhoon Trio+ Variable Mode Imager (GE Healthcare) (excitation nm, emission filter). (**C**) Voltage dependent recordings of KvAP currents at different voltages (Voltage scheme shown in blue lines). (**D**) Continuous single-channel records of KvAP currents at +100 mV on DOTAP bilayer and all point histogram of the current trace. (**E**) The current versus voltage (I-V) plots of the KvAP channel incorporated in DOTAP lipid bilayer. All recordings were done under symmetrical conditions; the medium contained 500 mM KCl, 10 mM HEPES, and pH 7.45.

We previously described the production and functional analysis of ion channels in a *Sf*21 batch based cell-free synthesis system^42^ receiving protein yields up to 8 µg/ml. To show the opportunity to produce high yields of an ion channel we wanted to demonstrate the synthesis of the ion channel KvAP in a CHO based CECF system. Quantification of produced KvAP resulted in protein yields approximately of 117 ± 9 µg/ml in the translation mixture, 32 ± 3 µg/ml in supernatant 1 and 66 ± 7 µg/ml in the microsomal fraction (Fig. 6A). It is reported that formation of multimeric structures can be detected using a SDS-PAGE in the case of stable formation of multimeric complexes under SDS-PAGE conditions^43^. Sackuchi *et al*. demonstrated a tetramer formation of influenza virus M2 channel on SDS-PAGE^44^, while Heginbotham et.al. showed a multimeric complex for a prokaryotic K+ channel^45^. In order to monitor protein mass and modifications including multimerizations we analyzed synthesized KvAP using SDS-PAGE followed by autoradiography and in-gel fluorescence (Fig. 6B). To enable in-gel fluorescence analysis, a radio label free detection method for proteins was established. Therefore, KvAP was synthesized in the presence of BODIPY-TMR-Lys tRNA_Phe_ which statistically incorporates into proteins using the phenylalanine anticodon. BODIPY-TMR-Lys tRNA_Phe_ was added to the reaction chamber at a final concentration of 2 µM at the beginning of the reaction. As expected, visualization by autoradiography and in-gel fluorescence led to distinct protein bands around 38 kDa and 62 kDa, whereas supernatant fraction only showed a faint signal. An additional protein band was visible in the in-gel fluorescence image at around 98 kDa. This evidence indicated the formation of multimeric structures required for KvAP functionality^46^.

Figure 6C shows the voltage dependent KvAP activity from the DOTAP planar lipid bilayer fused with KvAP-DOPC proteoliposomes. At a transmembrane voltage of −100 mV, there was a typical single-channel activity showing opening and closing transitions with a low conductance of around 140 pS at 500 mM KCl and when the voltage was depolarized to +100 mV, rapid transitions between distinct current levels were observed, consistent with the opening and closing of individual potassium channels with a conductance of, 340 pS at 500 mM KCl. In general, membrane bilayer showed far more channel events at +100 mV than at −100 mV. This suggests that the channels in the bilayer are voltage-gated and retain the functionality. At 0 mV there is no activity. Figure 6D shows the single channel recordings of the KvAP proteoliposomes fused to the DOTAP lipid bilayer at +100 mV. All-points amplitude histograms of the current trace at +100 mV fitted by Gaussian function show open (10 pA) and closed states (44 pA). Figure 6E shows the single channel current-voltage (I-V) curve plotted at different voltages. The I-V curve is nonlinear shows the protein inserted into the lipid bilayer and exhibits the voltage dependency. Negative control measurements were performed with proteoliposomes derived from the microsomes without any KvAP synthesis. In the case of the control, there was a capacitive fusion peak, but no pore formation and channel activity was observed. There were some cases of noisy, large irregular and unspecified currents during the whole process of electrophysiology measurements from both the negative control as well as the KvAP synthesized microsomes. These recordings were unstable and lasted for few seconds. We were never able to find a stable potassium channel recordings in our negative control measurements.

## Discussion

In this study we have demonstrated the possibility to combine a cell-free system based on CHO cell lysates and a continuous exchange technology for fast and efficient high yield production of proteins. We were able to show that prolonged reaction times lead to increased protein yields compared to a batch reaction due to semi continuous, passive energy delivery and removal of inhibitory byproducts. In general, the limitation of protein yield is caused by inhibition of the translational machinery which requires the presence of active translation factors. To circumvent these issues, we have performed CHO based CECF synthesis in a translation initiation factor independent manner using a cricket paralysis virus internal ribosomal entry site (CRPV IRES) which does not require ATP or GTP. Apart from that, factors necessary for translational elongation, termination and recycling of the protein synthesis machinery require energy components^47^. Factor eEF1A binds an aminoacyl-tRNA in a GTP dependent manner, directs the tRNA to the A site of the ribosome and triggers GTP hydrolysis after recognition of the cognate codon^48^. The second eukaryotic elongation factor eEF2 translocates the tRNA to the canonical E and P site while hydrolysis of GTP^49^. Additionally, performance of eukaryotic termination factors takes place while hydrolyzing GTP and recycling of protein translation complex requires ATP^50^. Therefore, long term delivery of energy during CHO CECF reaction enables prolonged activity of the translational machinery resulting in a significant increase of protein yields. The comparison of batch and CECF reactions for EGFR synthesis exemplarily showed a 40 fold increase in protein yield while having the same labor effort. When comparing batch and CECF reaction the increase in protein yield differs depending on the synthesized protein. This might depends on the efficiency of translation initiation. Therefore, variations in the protein encoding gene sequences might result in different protein yields. Gingold *et al*. reported that translation initiation is highly depending on the sequence content in the region of −15 to +18 of the gene sequence^51^. It is demonstrated that ribosome high-occupancy genes shows a pointed elevated usage of nucleotides C and U, in the 5th and 6th positions in the open-reading frame^51,52^. Due to the sequence differences of the synthesized proteins, which harbor various sequence contents, distinctions in translation efficiency and protein yield were detected. Due to the supply of the CECF feeding mixture, the effective costs for a typical CECF reaction are approximately 10 fold higher than the calculated costs for a typical batch reaction. Taking the achieved increase of protein yield into account, 4 times more amount of protein could be received for the same cost using a CECF reaction. One microgram of EGFR produced in a CHO based CECF reaction can be obtained for 0.4 USD or 0.35 Euro. This calculation is based on the expenses for total material requirement. For this, lysate preparation costs were determined based on the cultivation, raw lysate preparation and reconditioning costs. Moreover the costs for translation buffer (Mix A) and energy mix (Mix A) as well as plasmid preparation were taken into account.

Previous studies have demonstrated the activity of caspases in cell-free systems based on *Sf*21 lysates^40^. For optimization of CECF reaction based on CHO cell lysate we have tested different types of caspase inhibitors to detect the individual activity of these key regulators and thereby increase the protein production rate. Caspases are cysteine-dependent aspartate-specific proteases, key regulators of apoptotic processes and generally present as inactive zymogens in the cells^53,54^. Caspases can be activated by different types of cellular stress^55^. During lysate preparation, cells were stressed due to intensive washing procedures and mechanical disruption. Activated caspases cleave key regulators in protein translation process and lead to inhibition of protein translation^56^. In addition, cell-free produced proteins may be a target for cleavage through caspases^16^ as it was demonstrated in this study for the model protein EGFR. To circumvent the negative influence of active caspases on cell-free protein synthesis, specific inhibitors were tested during the CECF process. Previous studies have demonstrated the positive effect of the application of caspase inhibitors in mammalian cell culture on viability and target protein production yield^57^. The supplementation of caspase inhibitor AC-DEVD-CMK led to highest protein yields in CHO CECF system. AC-DEVD-CMK is an irreversible inhibitor of caspase-3 as well as caspase −6, −7, and −8^58,59^. The activity of previously named caspases seems to have a significant influence on the translational machinery of CHO lysates and constitutes one of the outstanding points for optimizing protein production in CHO based CECF systems.

With a view to cellular process and localization of transcription and translation there is a considerable difference between milieu conditions of both reactions. Adaptation of reaction conditions is therefore indispensable to obtain optimal conditions for coupled CECF reactions. Broedel et.al. performed studies to evaluate the optimal magnesium concentration in batch formatted cell-free synthesis reactions based on eukaryotic cell lysates^31^. They reported an optimal magnesium concentration of 3.9 mM for a CRPV IGR IRES dependent cell-free synthesis using CHO cell lysates^31^. Apart from that, Thomen *et al*.^60^ reported that velocity of T7 RNA polymerase depends on the concentration of magnesium in the transcription buffer. Maximum velocity of T7 RNA polymerase was reached by adding 20–30 mM magnesium^60^. In this study, we shifted the reaction conditions in the beginning of the CECF reaction to an increased magnesium concentration thus enabling optimal conditions for the T7 RNA polymerase in the first part of the reaction. For this, we supplemented magnesium into the reaction chamber in the beginning of the synthesis, which in turn led to increased protein yields. The obtained result indicates that production of high amounts of mRNA during the first part of the CECF reaction seems to be beneficial for target protein synthesis. The feeding chamber contains a reduced amount of 3.9 mM magnesium. Due to this concentration gradient, magnesium continuously diffuses from the reaction chamber to the feeding chamber. This again leads to a decrease of magnesium concentration in the reaction chamber depending on the reaction time. Finally the concentration of both chambers reaches an equilibrium. Magnesium balance results in a shift to optimum concentration of magnesium for CRPV dependent translation initiation which requires a magnesium concentration of 3–4 mM. These conditions enable the generation of a correctly folded secondary IRES structure needed for Cap independent translation initiation^61^. In conclusion, optimizing the magnesium concentration triggered a significant improvement of protein concentration in the CHO CECF system.

Apart from EGFR two further model proteins from different protein classes were tested in the CHO CECF system. It is well known that disulfide bridged proteins such as antibody fragments can be synthesized in various cell-free systems but reaction conditions need to be adjusted to preserved optimal redox conditions for correct folding of proteins^62,63^. Until now, antibody fragments were synthesized in a microsome containing eukaryotic cell-free system yielding up to 15 µg/ml^41,64^ in a batch based and up to 180 µg/ml^35^ in a CECF based system. In this study we have demonstrated a significant improvement of single chain protein yield up to more than 20 and 2.5 fold compared to *Sf*21 based batch and CECF system, respectively. This opens entirely new ways for further applications including the rapid bioengineering of tailor-made antibody fragments and fast development of single chain fragment libraries. Functional activity of single chain fragments in the microsomal fraction is higher than in the supernatant fraction. Similar behavior was reported for the *Sf*21 lysate based system^33^. Due to the fact that microsomal fractions of CHO lysate are derived from endoplasmic reticulum, microsomes may contain proteins including protein disulfide isomerase or binding immunoglobin protein. These proteins are essential for disulfide bridging and correct folding of disulfide bridged proteins^65^. Therefore endogenous microsomes present in the analyzed CHO lysates are of outstanding advantage when using the CHO CECF system. Finally, a 30 fold increase in functionally active protein was obtained in the CECF reaction compared to batch reactions. Addressing this fact, the CHO CECF system provides a platform for fast, high-yield and cost-effective synthesis of antibody fragments.

Cell-free synthesis and activity analysis of ion channels were tested. Ion channels in particular play a significant role in physiological processes. Alternative labeling using *BODIPY-TMR-Lys tRNA*_*Phe*_ was demonstrated which enables the possibility to detect proteins without using radio labels. Fluorescence labeling offers advantages concerning fast analysis of proteins using in-gel fluorescence and analysis of localization of *de novo* synthesized protein. Functionality of KvAP was demonstrated after synthesis in microsomes. The applied measurement is reduced to the analysis of single channel activity, therefore the total amount of functional protein cannot be quantified. The protein showed a clear and stable response for a long time at different voltages with a large conductance at 500 mM KCl. This single channel activity behavior of KvAP is quite characteristic like reported before^66^. Voltage dependence could be verified by using different lipid composition for proteoliposomes and planar lipid bilayer. Additionally this assay of cell-free synthesis and functional assessment could be adapted to a broad range of ion channel proteins.

The fractionation analysis revealed the presence of synthesized proteins in the supernatant fraction 1 for all produced proteins. This might have several reasons addressing the translocation of membrane proteins and secreted proteins into the microsomal fraction of the CHO cell lysate. Firstly, translocation into ER derived microsomal vesicles might be limited due to the limitation of translocation components. In *in vivo* protein production processes based on CHO cells several studies are available dealing with the optimization of translocation efficiency. Studies of Kober *et al*. analyzed exemplarily the efficiency of various signal peptides and showed differences in protein secretion^67^. Further studies of Le Fourn *et al*. demonstrated the positive effect of signal recognition particle overexpression on protein translocation, secretion and functionality^68^. These cellular bottlenecks might be a limiting factor for the translocation in cell-free systems as well and therefore represent a future target protein for the improvement of the CHO cell-free system. A reduced activity of EGFR and scFv was additionally detected in the supernatant fraction 1, which might give the impression that translocation is not required to obtain active proteins. The specific activity of EGFR in the microsomal fraction in comparison to the supernatant fraction seemed to be similar. In general, the hydrophobic character of membrane proteins might usually result in protein aggregation in a non-lipid containing environment. This leads to the assumption that small microsomes remain in the supernatant fraction after standard centrifugation procedure (16000 × g, 15 min, 4 °C), resulting in the presence of small EGFR containing microsomes in the SN1 fraction. To verify this observation, we have performed a particle measurement which indicates a reduced presence of 100 nm to 500 nm particles in the supernatant fraction 1 constituting the size range of the heterogeneous microsomes of the CHO cell lysate (supplementary figure 3). By performing a high-speed centrifugation protocol (50.000 × g, 60 min, 4 °C), the degree of EGFR-eYFP separation was improved, indicating a more successful partition of small microsomal particles (supplementary figure 1). Disadvantageous might be the formation of large microsomal aggregates leading to the assumption that further investigations are required concerning the separation protocol. Besides the presence of small microsomal structures in the supernatant fraction there is the possibility that soluble small complexes of EGFR can be formed due to the fact that hydrophobic EGFR only contains one transmembrane domain but large hydrophilic extracellular and intracellular domains.

Apart from the presence of proteins in the supernatant fraction, the microsomal fraction might not only contain translocated proteins but also membrane associated and aggregated proteins. Waldo and colleagues represent an assay for monitoring the aggregation and folding status of proteins by fusion of a fluorescent GFP tag to the C-terminus of a target protein, while showing that folding of a protein correlates to the derived fluorescence signal^69^. It was reported that the formation of the chromophore of GFP depends on the correct folding of the protein, which can be transferred to the folding and fluorescence activity of the related protein eYFP. In this study, the fusion protein EGFR-eYFP showed a high fluorescence signal on µ-ibidi slides and during CLSM analysis, which indicates the presence of correctly folded eYFP fusion proteins. The CLSM analysis underlines the partly location of EGFR-eYFP in the microsomes of the CECF reaction, which might be due to the translocation of the protein and embedding of EGFR into the membrane. The presence of translocated protein was further investigated by the analysis of protein glycosylation using radio labeled mannose. A prerequisite for protein glycosylation is the translocation of the protein into the lumen of the ER based microsomes. The detection of a radio labeled EGFR protein band in the autoradiograph (supplementary figure 5), which disappears after glycosidase treatment (EndoH, PNGaseF) and is not present in the control sample without EGFR encoding DNA (NTC), supports the assumption of the presence of translocated EGFR proteins. Passive, posttranslational integration, without the help of chaperons and translocons present in the microsomal membrane, and delivery of EGFR from the cytosolic surrounding to the microsomal fraction can be excluded due to results from “fluorescence recovery after photo bleaching (FRAP) experiments” (supplementary figure 6A) and analysis of passive integration of EGFR-eYFP produced in a microsome depleted lysate into “post-protein-production” supplemented microsomes (supplementary figure 6B). FRAP experiments revealed no fluorescence recovery of previously bleached microsomes after 60 s of incubation thereby indicating the low potential of passive diffusion of EGFR-eYFP into microsomal membranes. Apart from that, addition of microsomes to a sample containing produced EGFR-eYFP in microsome depleted CECF reaction results in no passive integration of protein into the microsomal fraction. The combination of the obtained results gives a hint that a high amount of EGFR-eYFP is translocated into the microsomes of CHO CECF lysate, whereas partly aggregation and membrane association of EGFR-eYFP cannot be completely excluded. Apart from that, a passive integration of EGFR during translational process due to the absence of translocation components including the SRP cannot be excluded by the obtained results. The discussed bottlenecks concerning protein translocation, protein aggregation and membrane association can be transferred to the different types of proteins. As exemplarily demonstrated for EGFR, translocation of proteins was detected, but the presence of a heterogenic mixture of conformational differing proteins cannot be excluded. This bottleneck already started with heterogenic character of the applied cell line, which is already reported for *in vivo* protein production processes^70^. Future evaluations starting from the cell line development up to adaptation of protein production conditions are required to analyze and improve the quality of produced protein.

In summary, we have demonstrated the high yield production of various classes of proteins including the transmembrane protein EGFR, a single chain variable fragment and the ion channel KvAP thereby showing the diversity and the opportunities connected to CHO CECF systems. Nowadays, various continuous exchange cell-free systems are available originating from *E*. *coli* ^21^, wheat germ^14,71^ and *Sf*21^40^ cells enabling the synthesis of high amounts of protein. CECF systems based on *E*.*coli* and wheat germ are useful for the synthesis of non-modified proteins but these systems are often disadvantageous for proteins of mammalian origin that are mostly required for drug development studies. *Sf*21 and CHO CECF systems offer opportunities for the synthesis of complex and difficult-to-express proteins due to their eukaryotic origin and their endogenous microsomal structures mimicking the natural milieu of the proteins. In comparison to the *Sf*21 CECF system^16,35^, production of the target proteins in CHO CECF system enables an increase in production efficiency reaching protein yields up to around 950 µg/ml. The CHO CECF system is based on a prominent industrial production host. CHO cells are well-established for recombinant protein production for more than 3 decades and a continuous improvement concerning cell line development, productivity, media development and stability is achieved^5,72^. According to these developments CHO cells provide an optimal basis for the development of a CECF system. The CHO CECF offers additionally an opportunity for prescreening of DNA templates for industrial approaches. This might lead to a time reduction by avoiding extensive selection of CHO cell clones and might result in future improvement for the industrial protein production pipeline.

Various studies are currently available dealing with reactor design for the cell-free synthesis. Miniaturization and microfluidic devices constitute a valuable approach to enable high throughput setups and minimize reagent volumes and costs. The adaptation of *E*.*coli* CECF systems to these devices is already reported^73,74^. The joining of the technology with the developed the CHO CECF system might be an advantage for future high throughput approaches.

The demonstrated achievements are a beneficial basis for further applications, including NMR studies, ligand binding studies as well as inhibitor screening assay. Previous studies of Bocharova *et al*.^75^ and Proverbio *et al*.^76^ already showed the synthesis and structural estimation of membrane proteins using E.coli CECF system. The development of the CHO CECF system might lead to an improvement in the structure determination of mammalian membrane proteins due to the eukaryotic nature of the system. It is reported that automation^77,78^ and high throughput screenings (HTS)^62,79^ are possible applications for cell-free protein synthesis systems. The origin of the novel CHO CECF system is compatible to the majority of industrial drug protein production processes and advantageous for adaptation to drug development line. Combination of these features will provide outstanding opportunities for faster and more efficient evaluation, variation and production of pharmaceutically relevant protein targets.

## Methods

### Generation of DNA templates

The following model proteins were selected for evaluation of CHO lysate based CECF system: The enhanced yellow fluorescent protein (eYFP), a soluble reporter protein, the epidermal growth factor receptor (EGFR), a transmembrane protein receptor, the voltage-gated potassium channel (KvAP), an ion transport channel and the single chain antibody variable fragment SH527-IIA4, an antibody fragment binding SMAD2-P antigen harboring a c-terminal c-myc tag^41,80,81^. Regulatory sequences essential for cell-free protein synthesis were fused to 5′ and 3′ UTR of the genes using expression PCR^82^. 5′ UTRs contained a T7 promotor sequence and an internal ribosomal binding site (IRES) of the Cricket Paralysis virus intergenic region (CRPV). Native signal sequences were substituted by the melittin signal sequence (Mel). 3′UTR of all DNA templates contain a T7 terminator sequence. After successful two-step expression PCR, linear DNA fragments were cloned into a pIX3.0 vector (Biotech Rabbit) backbone. The integrity of all constructs was verified by sequencing. Plasmids were transformed in *E*.*coli* JM109 and isolation was carried out using GeneJET Plasmid Miniprep Kit (Thermoscientific). The batch based cell-free synthesis was performed with identical DNA templates as used for the CECF system.

### Cell-free protein synthesis in batch and CECF format

In general, cell-free protein synthesis based on CHO cell lysates required three different mixes. The basis for cell-free protein synthesis is provided by the CHO lysate mix. Preparation of CHO lysate containing endogenous microsomal structures was described in previous studies^26,83^. The lysate is treated with S7 nuclease to remove endogenous RNA and supplemented with an energy generation system based on 100 µg/ml creatine kinase (Roche) and bulked tRNA from bakers yeast (Roche). 40% of lysate mix was applied to cell-free protein synthesis reaction. The second component, Mix A (10x), was composed of 300 mM HEPES-KOH (pH 7.6), 2250 mM KOAc, 2.5 mM spermidine, 1 mM of the 20 standard amino acids each (Merck) and 39 mM Mg(OAc)_2_. The third component Mix B (5x) consisted of 100 mM creatine phosphate, 8.75 mM ATP, 1.5 mM CTP, 1.5 mM UTP, 1.5 mM GTP (Roche). Additional components were supplemented to the cell-free synthesis reaction depending on the reaction mode. A standard 50 µl batch reaction contained 20 µl lysate mix, 5 µl Mix A, 10 µl Mix B, 0.5 mM m7G(ppp)Gcap analogue (Prof. Edward Darzynkiewicz, Warsaw University, Poland), 120 ng/µl plasmid DNA, a final concentration of 1 U/µl T7 RNA polymerase (Stratagene). Reactions were performed in an 1.5 ml Eppendorf tube. To fill up to a defined volume, RNase free water was added to the reaction. To start the cell-free reaction, the readily prepared sample was placed in a thermomixer for 3 h (Eppendorf) at 30 °C and 600 rpm. A standard CECF reaction consisted of a reaction mix (50 µl) and a feeding mix (1000 µl). Reaction mix was constituted like a batch reaction, but without adding m7G(ppp)G cap analogue. In addition, sodium azide was supplemented to reaction to prevent microbial growth during cell-free synthesis. The feeding mix contained 1x Mix A, 1x Mix B and 0.02% of sodium azide. CECF synthesis was performed in a commercially available two chamber dialysis device (RiNA GmbH, catalogue number SVC100) for 48 h and 600 rpm in a thermomixer (Eppendorf). In both reaction formats radio labeling of synthesized proteins was achieved by incorporation of ^14^C leucine (Perkin Elmer). Concentrations of 60 µM ^14^C leucine for the batch reaction (specific radioactivity 66.67 dpm/pmol) and 11 µM ^14^C leucine (specific radioactivity 9.9 dpm/pmol) for the CECF format were supplemented to enable further qualitative and quantitative analysis of cell-free synthesized proteins.

### Quantification of cell-free synthesized proteins

After finishing cell-free reactions, yields of radio labeled proteins were determined by hot TCA precipitation followed by liquid scintillation measurement as described previously^33^. For fractionation of translation mixture (TM) into supernatant 1 (SN1) and microsomal fraction (MF), batch or CECF sample was centrifuged for 15 min at 16000 × g and 4 °C. Pelleted fractions containing the microsomes and were resolved in the corresponding volume of PBS. Additionally, a high speed centrifugation for evaluation of centrifugation procedure was performed for 60 min at 50000 × g and 4 °C.

### Denaturing PAGE, autoradiography and glycosylation analytic

For separation of proteins by SDSPAGE, 2.5 µl (CECF) or 5 µl (batch) of the desired fraction (translation mixture, supernatant 1, microsomal fraction) was precipitated by adding of 150 µl ice cold acetone. Acetone precipitation was performed as described previously^25^ and protein pellets were resolved in LDS sample buffer (Thermoscientific) including 50 mM of DTT. Protein samples were separated on NUPAGE 10% Bis-Tris gels or 3–8% Tris-Acetate gels (for EGFR samples) (Thermoscientific). For autoradiography analysis, protein containing gels were dried using a vacuum chamber (Unigeldryer 3545D, Uniequip) for 1 h and 70 °C and deposited on a phosphoscreen. Phosphoscreen was scanned using a variable mode imager (Typhoon TRIO + Imager, GE Healthcare) to receive autoradiography of radiolabeled proteins.

To enable an initial assessment of protein glycosylation, radio labeled ^14^C mannose (American Radiolabeled Chemicals Inc) was directly added to the cell-free reaction for incorporation into the target protein. A final concentration of 3 nM ^14^C mannose was directly supplemented to the reaction and feeding chamber of the CECF reaction. To verify the glycosylation of target protein, the microsomal fraction of the produced protein was treated with glyco-residue removing enzymes PNGaseF and EndoH according to the manufactures protocol. Proteins were prepared for SDS-PAGE separation as described previously and separated and analyzed according to the standard protocol.

### Fluorescence analysis

eYFP fusion proteins (eYFP and Mel-EGFR-eYFP) were analyzed on a µ-Ibidi slide using a variable mode imager (Typhoon TRIO + Imager, GE Healthcare) with an excitation wavelength of 488 nm and an emission wavelength of 526 nm. 5 µl of sample was diluted in 20 µl of PBS for further analysis of fluorescence detection and quantification. Graphical analysis of fluorescence images was performed by application of Image Quant TL software (GE Healthcare). Microscopic images of EGFR-eYFP were taken on a LSM 510 meta (Zeiss) confocal laser scanning microscope (CLSM). For analysis of fluorescent eYFP, protein was excited at a wavelength of 514 nm and resulting emission signal was detected using a meta detector >529 nm. Photo bleaching was performed using an argon laser at 488 nm with 100% laser intensity and 50 iterations. Images of bleached samples were taken after 0 s, 15 s, 30 s, 45 s, 60 s after inducing bleaching.

Statistical fluorescence labeling of KvAP was accomplished by supplementation of phenylalanine anticodon tRNA harboring the non-canonical amino acid BODIPY-TMR-Lys. BODIPY-TMR-Lys tRNA_Phe_ was added at a final concentration of 2 µM during CECF reaction. Samples were separated on 10% Bis-Tris PAGE gels (Thermoscientific) followed by an incubation in 50% methanol/water solution for 30 min. In-gel fluorescence signal was detected using a variable mode imager (Typhoon TRIO + Imager, GE Healthcare) and an excitation wavelength of 532 nm followed by an emission wavelength of 580 nm.

### Tyrosine Kinase Assay

To detect autophosphorylation activity of EGFR 15 µl of microsomal fraction were pelleted by centrifugation (16000 × g, 10 min, 4 °C) and supplemented with 300 µl kinase buffer composed of 100 mM HEPES (pH 7.4), 1% glycerol, 0.1 mg/ml BSA, 5 mM MgCl_2_, 1.25 mM MnCl_2_, 0.1 mM NaVO_3_, 2 µM caspase inhibitor and 200 µM ATP. Microsomal fraction resolved in kinase buffer was incubated for 10 min at room temperature to enable phosphorylation reaction. Kinase buffer treated samples were precipitated by addition of 900 µl ice cold acetone. For analysis of translation mixture and supernatant 1 900 µl of kinase buffer were directly added to 15 µl of each fraction. With the intention to show specificity of autophosphorylation, control samples were prepared, treated with calf intestinal phosphatase (CIP) (New England Biolabs) for 10 min at RT to remove phosphorylation modification of EGFR. Therefore, pelleted microsomal fraction was resolved in 14 µl PBS followed by supplementation of 1 µl CIP. CIP treatment was followed by kinase treatment or directly by acetone precipitation depending on the sample application. Autophosphorylated EGFR samples were further separated on 3–8% Tris-Acetate gels (Thermoscientific) and transferred to a PVDF membrane using iBlot Device (Thermoscientific). The membrane was blocked in TBS + 0.1% Tween + 2% BSA for 2 hours and subsequently incubated with “Phospho-EGF Receptor (Tyr1068) (D7A5) XP® Rabbit mAb” primary antibody diluted 1:1000 overnight at 4 °C. Secondary “Anti-rabbit IgG, HRP-linked Antibody 7074” diluted to 1:2000 was added to enable detection of phosphorylated EGFR. Detection was carried out using the “Amersham ECL Prime Western Blotting Detection Reagent” (GE Healthcare) and the “Typhoon Trio + Variable Mode Imager” (GE Healthcare). After detection blotting membranes were dried and subjected to autoradiography.

### Functional analysis of single chain antibody variable fragment Mel-SH527-IIA4 by ELISA

Supernatant fraction 1 (SN1) and supernatant fraction 2 (SN2) containing cell-free synthesized Mel-SH527-IIA4 single chain fragment were analyzed for their binding activity by ELISA. Supernatant fraction 1 was prepared as described above. For preparation of supernatant fraction 2, microsomal fraction of cell-free reaction sample was treated with PBS + 0.2% n-Dodecyl-maltoside (DDM) and agitated for 45 min at room temperature to release translocated single chain fragments. SN2 fraction was separated by centrifugation from DDM treated microsomes (15 min, 16000 × g, 4 °C). ELISA was performed in a 96 well plate (Costar 96, Sigma-Aldrich) starting with streptavidin coating (0.74 μg/mL in PBS, 270 μL/well; Serva) for 1 h at RT and afterwards 2× washing in PBS supplemented with 0.05% Tween-20 (PBST). This step was followed by blocking of the plate overnight at 4 °C using PBS + 2% BSA and 3 times washing with PBST. 200 ng of biotinylated SMAD2-P antigen (Biotin-PEG-(GGS)2GPLQWLD-KVLTQMGSPSVRCSpSMpS) (Peps4LSGmbH, Heidelberg, Germany) diluted in PBS were supplemented to each well to enable further Mel-SH527-IIA4 binding. Following 3 times washing with PBST SN fractions of cell-free synthesized Mel-SH527-IIA4 were prediluted to a concentration of 0.5 ng/ml (16.55 nM) in PBST + 1% BSA, further serially diluted in 1% BSA in PBST and incubated for 1.5 h at RT. For detection of bound Mel-SH527-IIA4, wells were incubated with the monoclonal anti-c-myc-tag antibody 9E10 as primary antibody (Thermofisher; dilution 1:1000 in 1% BSA in PBST, 100 μL/well) and secondary anti-mouse-IgG-HRP-linked antibody (Cell signaling) (dilution 1:2000 in 1% BSA in PBST, 100 μL/well) for 1.5 h at RT, followed by three PBST washing steps. Final detection was accomplished by addition of 100 µl TMB solution (3,3,5,5-tetramethylbenzidine, 100 μL/well; Life technologies), further incubation while gently shaking for 15 min. Reactions were stopped by addition of 0.5 M sulfuric acid. The signal of each well was analyzed using a plate reader (FLUOstar Omega, BMG LABTECH). Absorbance was measured at 620 nm (scattered light) and 450 nm and values were subtracted from each other (450 nm–620 nm). To estimate background binding, translation mixture without added template (NTC) as well as PBS + 1% BSA (PBS) were analyzed in parallel.

### Functional assessment of KvAP

Proteoliposomes were formed from the KvAP synthesized microsomes. After translation followed by centrifugation, washing and resuspending the vesicular fraction with PBS, 25 µl of the solution containing KvAP harboring microsomes was mixed with 50 µL of 15 mM DOPC dissolved in buffer containing 200 mM cholate. After mixing for 30 min at room temperature, detergent was removed by Bio-Beads SM-2 (Bio-Rad) treatment. After discarding the biobeads, remaining solution containing the proteoliposomes was collected and used for the electrophysiology measurements. After formation of the lipid bilayer, 2–5 µl of the proteoliposomes were added to the planar lipid bilayer and currents were measured.

Planar lipid bilayers were formed from 1,2-dioleoyl-3-trimethylammonium-propane (Chloride salt) (DOTAP) (Avanti Polar Lipids, Albaster, AL,USA) over a 50 µm cavity on a 16 microelectrode array. Lipids were dissolved in octane (Sigma Aldrich, Munich, Germany) 2 mg/mL. Salt concentration of 500 mM KCl (Sigma Aldrich (Fluka), Munich, Germany), 10 mM HEPES, buffered at pH 7.45 was used as an electrolyte. Functional measurements of the KvAP channel were conducted using planar bilayers formed by DOTAP lipids. The cavity contains the nonpolarizable working electrode containing Ag/AgCl layer deposited on the underlying Cr/Au layer^84^. Briefly, 200 µl of electrolyte solution was added to the measurement chamber of an Orbit 16 System (Nanion Technologies GmbH, Munich, Germany). Planar bilayer was formed from the octane suspended DOTAP lipids^66,84,85^. A single channel amplifier (EPC-10, HEKA Electronic Dr. Schulze GmbH, Lambrecht, Germany) was connected to the multiplexer electronics port of the Orbit16 system. Recordings were done at a sampling rate of 50 kHz with a 10 kHz Bessel filter. Data were analysed with Clampfit (Molecular Devices, Sunnyvale, CA, USA).

### Estimation of particle size distribution in a cell-free reaction

To verfify the presence of microsomes in the translation mixture and supernatant 1 fraction of CHO cell-free reaction particle size distribution measurements were performed using a Zetasizer Nano ZS (Malvern Instruments Ltd). For this, supernatant fraction 1 was prepared according to the standard centrifugation protocol (16000 × g, 15 min, 4 °C). Translation mixture as well as supernatant fraction 1 were diluted 1:5 with PBS prior particle measurement. The software setup included a refractive index of 1.33 and a temperature setting of 25 °C. The obtaines values were calculated from three readings of three independent measurements.

## Electronic supplementary material

Supplementary Information
