# Supplementary material for: High-yield production of “difficult-to-express” proteins in a continuous exchange cell-free system based on CHO cell lysates
Source: Sci Rep. 2017 Sep 15;7:11710. doi: 10.1038/s41598-017-12188-8 (PMC5601898; doi:10.1038/s41598-017-12188-8)
Supplement: Supplementary file 1 — Supplementary Information [file 41598_2017_12188_MOESM1_ESM.doc]

**Supplementary Dataset**

**High-yield production of “difficult-to-express” proteins in a continuous exchange cell-free system based on CHO cell lysates**

Lena Thoring^1,2^, Srujan K. Dondapati^1^, Marlitt Stech^1^, Doreen A. Wüstenhagen^1^, Stefan Kubick^1^

^1^ Fraunhofer Institute for Cell Therapy and Immunology (IZI), Branch Bioanalytics and Bioprocesses (IZI-BB), Am Mühlenberg 13, D-14476 Potsdam, Germany

^2^ Institute for Biotechnology, Technical University of Berlin (TUB), Gustav-Meyer-Allee 25, 13355 Berlin

**Supporting information**


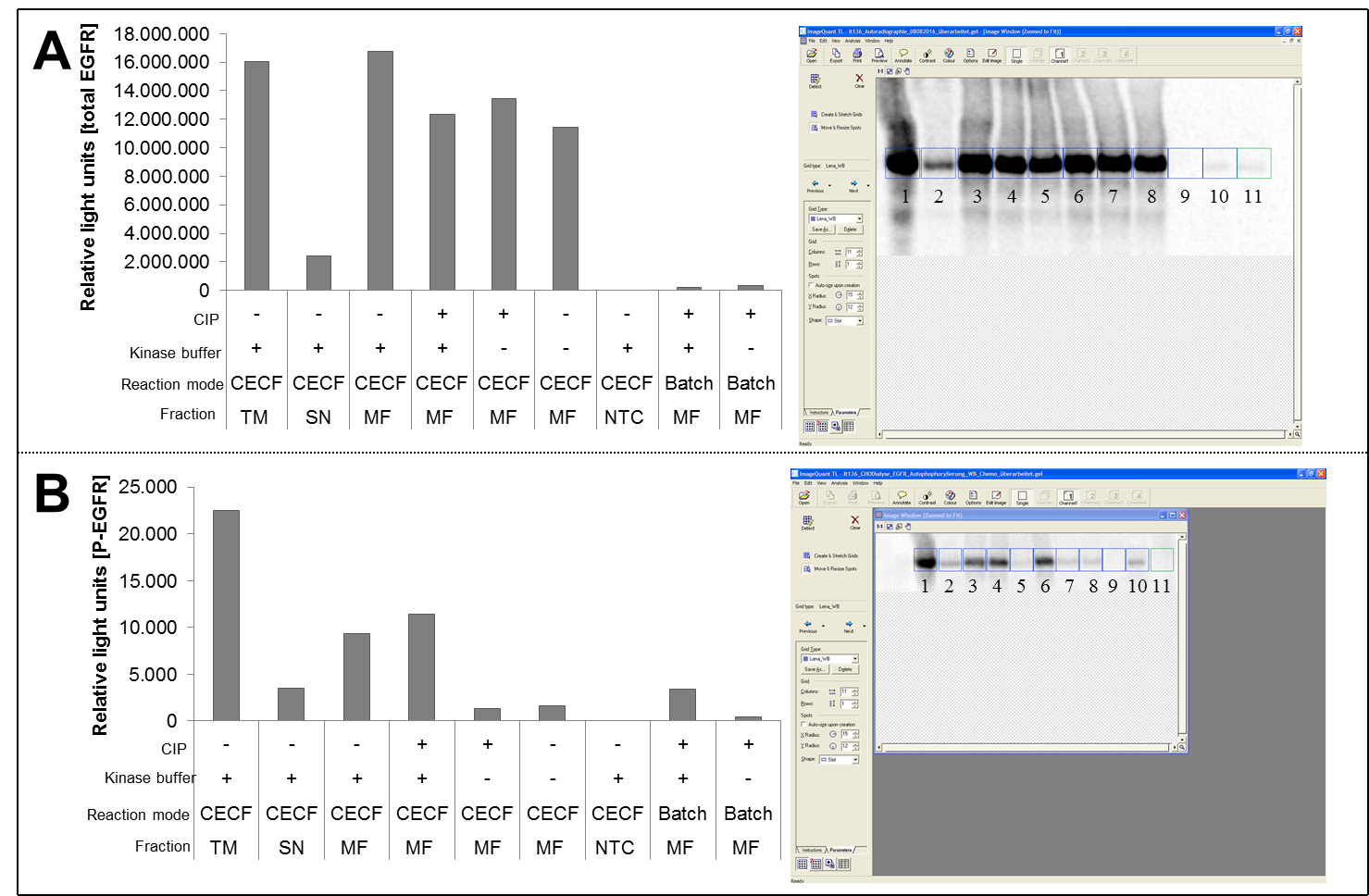


***Supplementary figure 1: Quantification analysis of in vitro phosphorylation of Mel-EGFR-eYFP tyrosine 1068 residue by immunoblotting and image analysis.*** *CECF samples were separated into translation mixture (TM), supernatant 1 (SN) and microsomal fraction (MF). To verify the specificity of the phosphorylation by EGFR kinase, samples were treated with calf intestinal phosphatase (CIP) to enable dephosphorylation of EGFR prior kinase buffer treatment. No template control (NTC) contained CHO cell-free translation mixture without supplementation of DNA template. (****A)*** *Graphical evaluation and quantification of total EGFR (left) based on the image analysis of protein bands on the autoradiography (right) using Image Quant TL software (GE Healthcare). Blue squares on the autoradiography indicate the area used for protein band intensity calculation (****B).*** *Graphical evaluation and quantification of phosphorylated EGFR (P1068) (left) obtained from image analysis of the western blot (right). Blue squares on the western blot indicate the area used for protein band intensity calculation. Sample number and description: 1. CECF TM + Kinase Buffer; 2. CECF TM + Kinase Buffer; 3. CECF MF + Kinase Buffer; 4 and 6. CECF MF + Kinase Buffer + CIP; 5. and 7. CECF MF + Kinase Buffer; 8. CECF MF; 9. NTC + Kinase Buffer; 10. Batch MF + Kinase Buffer + CIP; 11. Batch MF + CIP*


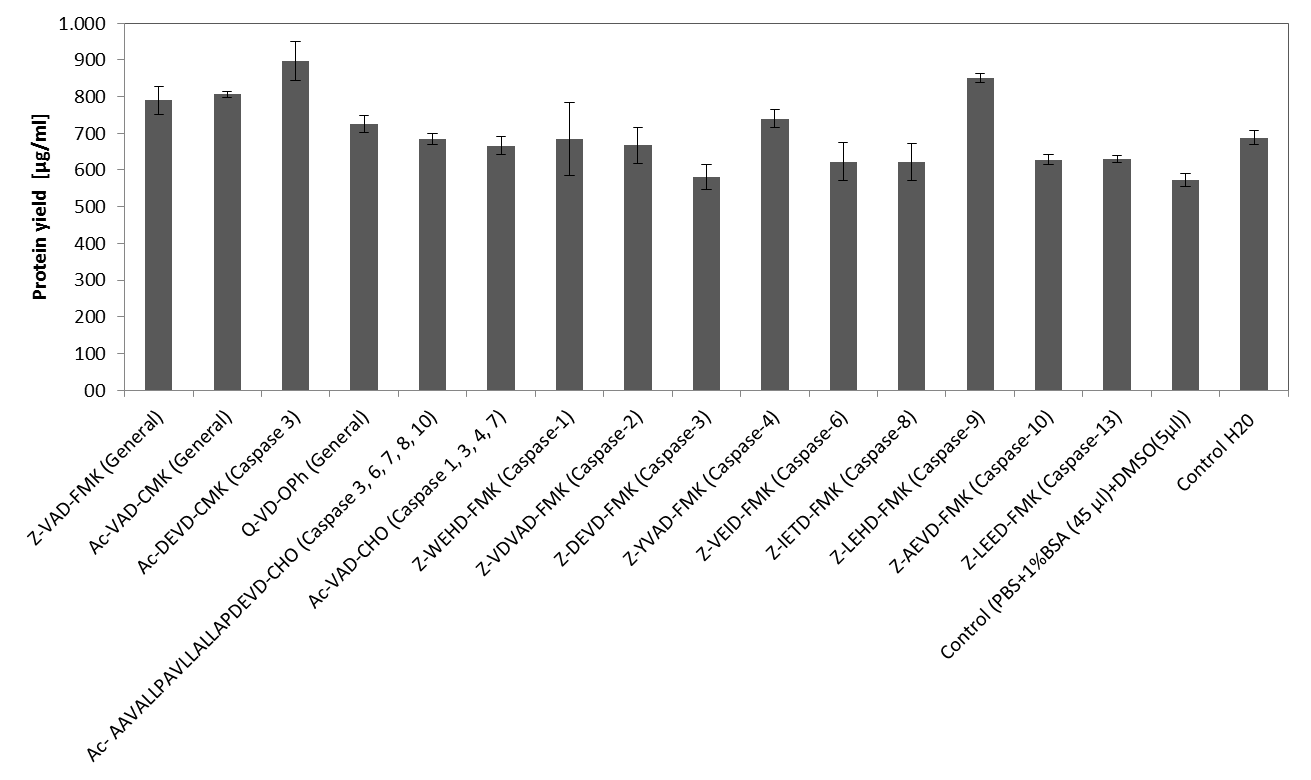


***Supplementary figure 2: Supplementation of various caspase inhibitors to CECF reaction.*** *Z-VAD-FMK, Ac-VAD-CMK, Ac-DEVD-CMK, Q-VD-OPh, Ac-AAVALLPAVLLALLAPDEVD-CHO, Ac-VAD-CHO, Z-WEHD, ZVDVAD-FMK, Z-DEVD-FMK, Z-YVAD-FMK, Z-VEID-FMK, Z-IEDT-FMK, Z-LEHD-FMK, Z-AEVD-FMK were supplemented to cell-free synthesis of Mel-EGFR. Protein yield was quantified by scintillation measurement of radio labeled proteins. Two controls were prepared including the addition of PBS+1%BSA and H_2_O instead of caspase inhibitor. Error bars represent the standard deviation of triplicate analysis.*

| *Protein name/ description* | *Gene name* | *Molecular weight [kDa]* | *Maximum protein yield [µg/ml]* | *Reference* |
| --- | --- | --- | --- | --- |
| Epidermal growth factor receptor | EGFR | 134.27 | 982.4 | Uniprot P00533 |
| Voltage-gated potassium uptake system KvaP | KvAP | 30.89 | 116.9 | Uniprot Q8EAX3 |
| Single chain fragment against SMAD-P | SH527-IIA4 | 30.2 | 396.9 | Stech et.al. 2014  Hust et.al. 2011  Schirrmann et.al. 2010 |

***Supplementary table 1:*** ***General information and total protein yields of EGFR-eYFP, KvAP and SH527-IIA4 obtained using CHO CECF system.***

*
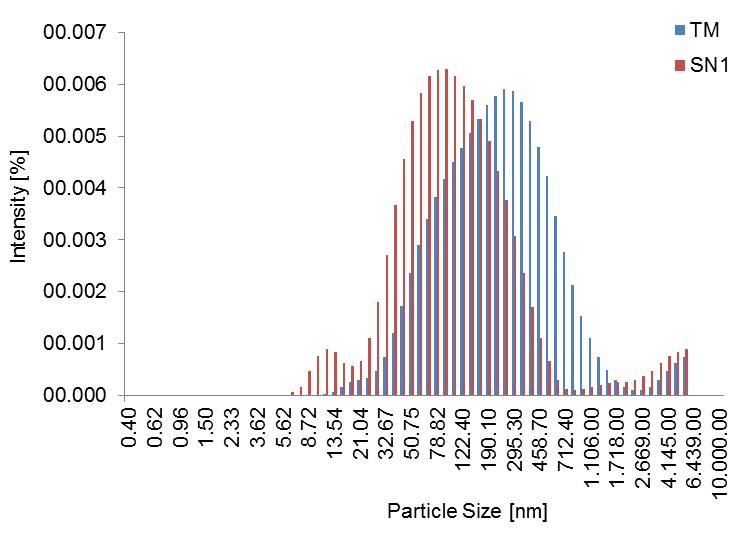
*

*Supplementary figure 3: Analysis of particle distribution in the translation mixture (TM) and supernatant fraction 1 (SN1) of the CHO cell-free reaction.* Particle light scattering intensity (intensity (%)) was measured using a Zetasizer Nano ZS (Malvern Instruments Ltd). Supernatant fraction was prepared according to the standard centrifugation protocol (16000xg, 15 min, 4°C). Prior to the measurement both fractions were diluted 1:5 with PBS and 15 µl of suspension was analyzed in a low volume glass cuvette. For the settings a refractive index of 1.33 was chosen according to the selection dilution solution of the sample. The obtained values were calculated from three readings of three independent measurements.


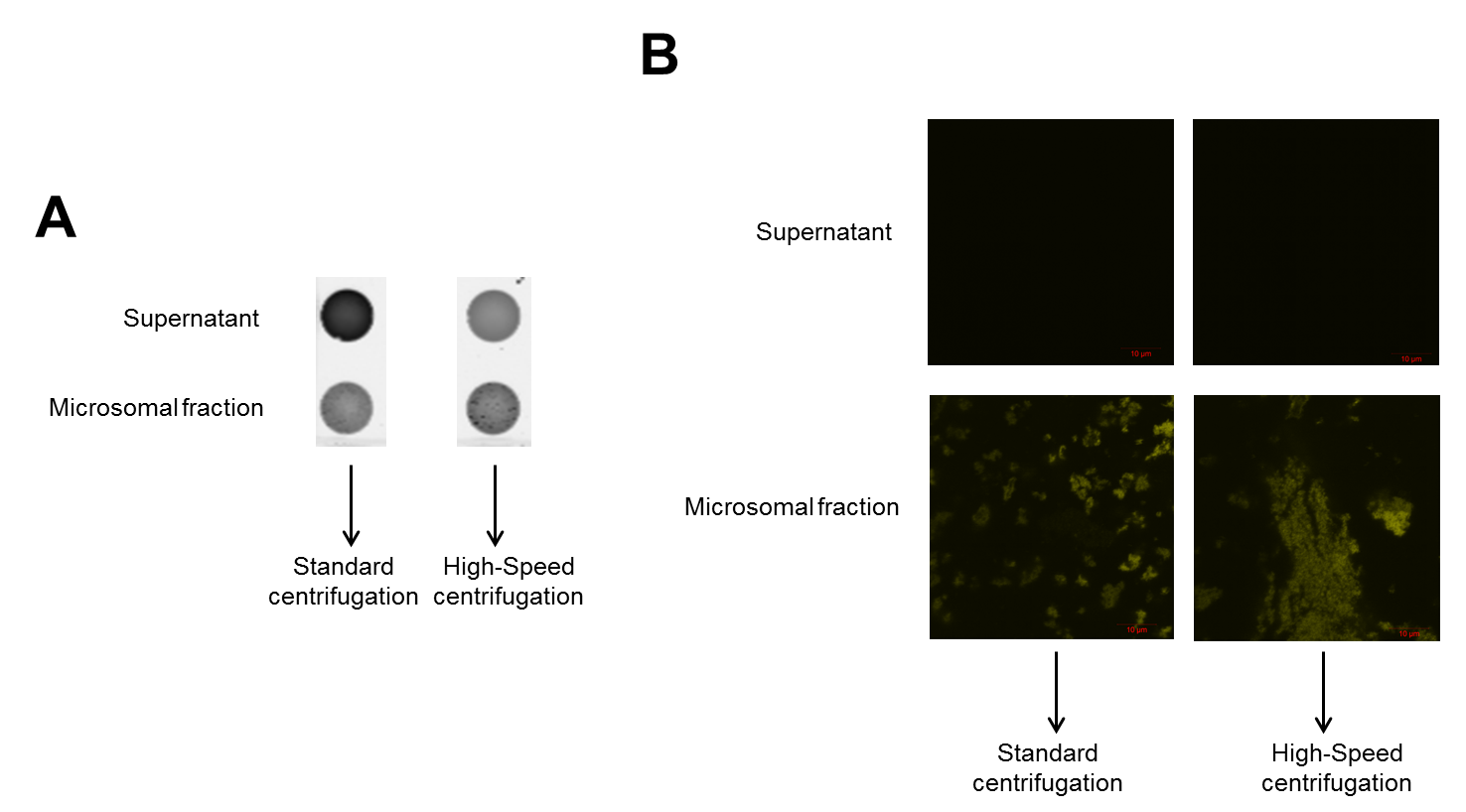


**Supplementary figure 4: Analysis of strategies for separation of microsomal fraction from CHO CECF reaction.** Translation mixture containing produced Mel-EGFR-eYFP was centrifuged using standard conditions (16.000xg, 15 min, 4°C) and high-speed conditions (50.000xg, 60 min, 4°C). (**A**) Separation efficiency was analyzed on a µ-ibidi slide by evaluation of fluorescence signal in the supernatant and microsomal fraction of CECF reaction. (**B**) A microscopic image of both fractions was taken after centrifugation (standard, high-speed) to visualize the localization of Mel-EGFR-eYFP and the appearance of microsomes after different centrifugation procedures.

**
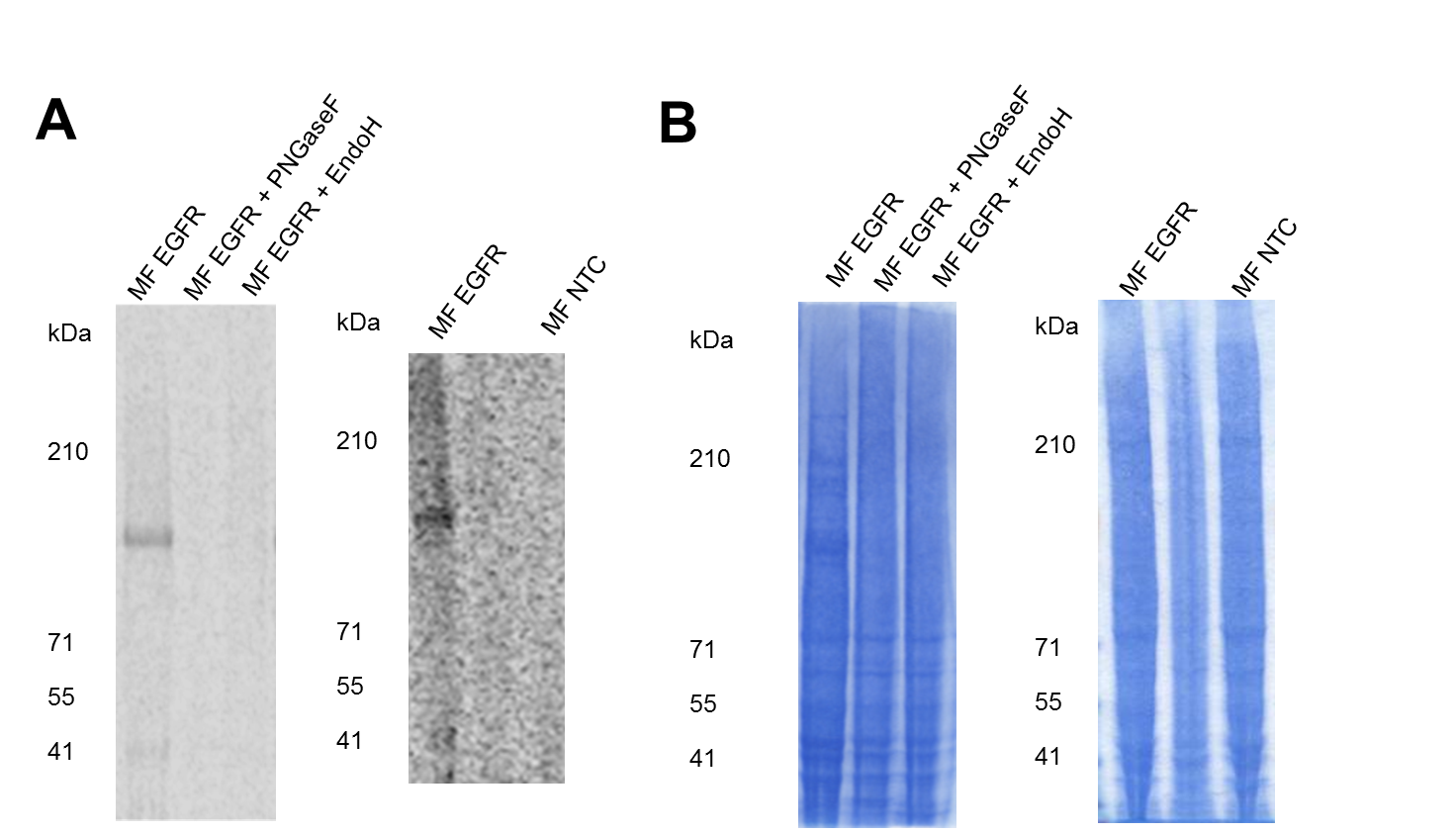
**

**Supplementary figure 5: Evaluation of EGFR glycosylation by incorporation of ^14^C Mannose followed by glycosidase treatment.** Mel-EGFR-eYFP was synthesized in the presence of ^14^C mannose to incorporate radio labeled sugar moieties. To evaluate posttranslational glycosylation of EGFR, microsomal fraction (MF) was treated with glycosidase PNGaseF and EndoH to cleave of sugar residues. Samples were separated on SDS-PAGE, coomassie stained (**B**) and analyzed on an autoradiogram (**A**). An appropriate glyco protein band was detected on the autoradiogram indicating the presence of posttranslationally modified EGFR. The specificity of the EGFR protein band was underlined by the analysis of a control without the EGFR encoding template DNA (NTC) showing no apparent protein band.


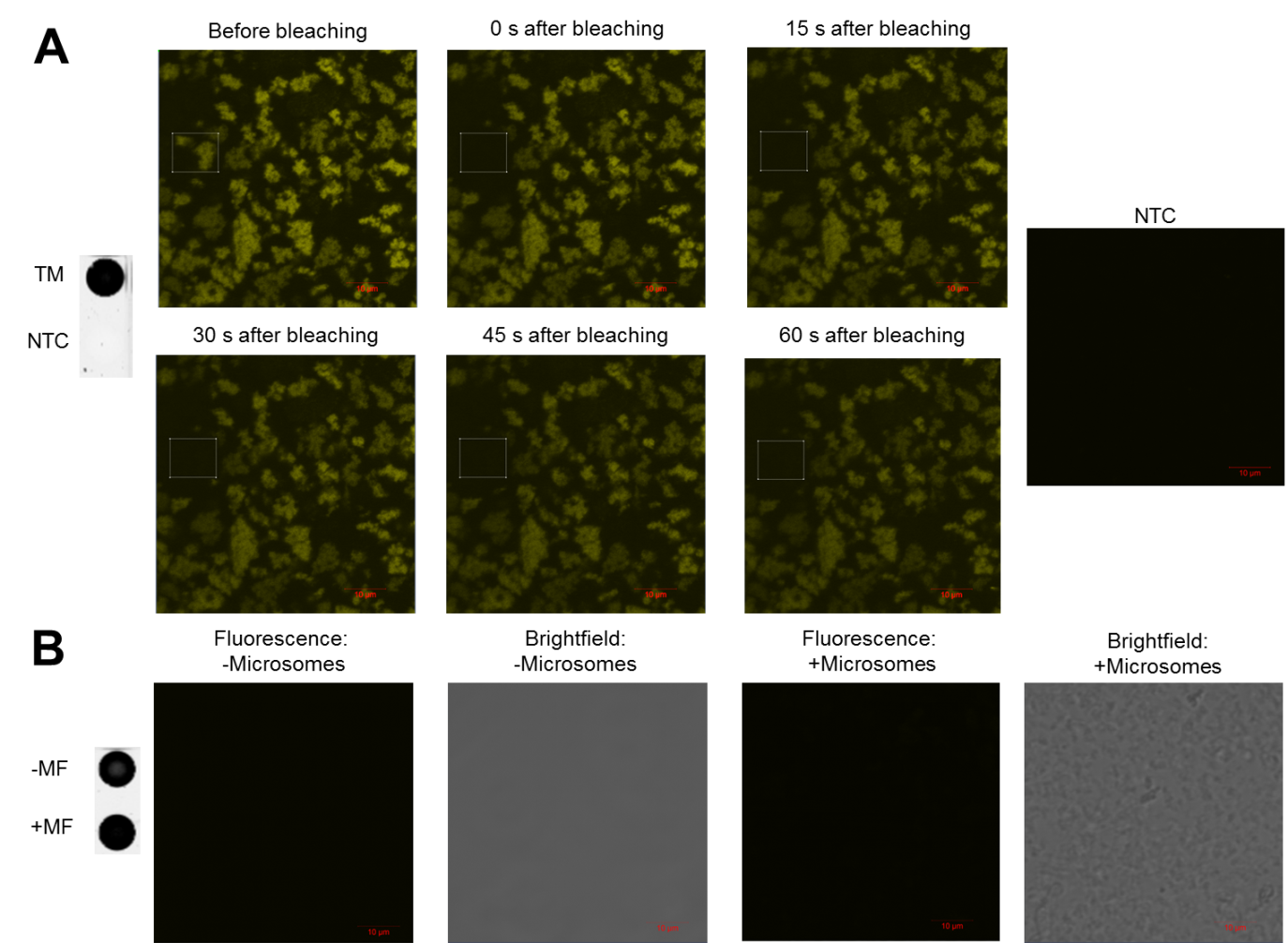


**Supplementary figure 6: Analysis of the integration of membrane protein EGFR into microsomal membranes (Frap analysis of membrane embedded proteins). (A)** Photobleaching of Mel-EGFR-eYFP present in the translation mixture (TM) of a CHO CECF reaction. No template control (NTC) contains a translation mixture without supplementation of DNA template. Fluorescence intensity of the samples was evaluated on an µ-ibidi slide prior performing the bleaching assay and indicated the presences of fluorescent proteins. Bleached area is marked on the microscopic images by a grey square. Microscopic images were taken before bleaching and 0 s, 15 s, 30 s, 45 s and 60 s after bleaching to monitor fluorescence recovery. No fluorescence recovery was detected 60 seconds after bleaching indicating no passive integration and membrane association of Mel-EGFR-eYFP into microsomes from the cytosolic surrounding. **(B)** Synthesis of Mel-EGFR-eYFP in a microsome depleted CHO CECF reaction followed by analysis of reintegration of Mel-EGFR-eYFP after supplementation of microsomes (MF) and four hours of incubation. Analysis of produced proteins on a µ-ibidi revealed the presence of fluorescent Mel-EGFR-eYFP. Supplementation of microsomes does not lead to a detection of fluorescence signal localized in the microsomal fraction. This underlines the assumption that no detectable passive integration of Mel-EGFR-eYFP from the cytosolic environment takes place. (Scale bar 10 µM)


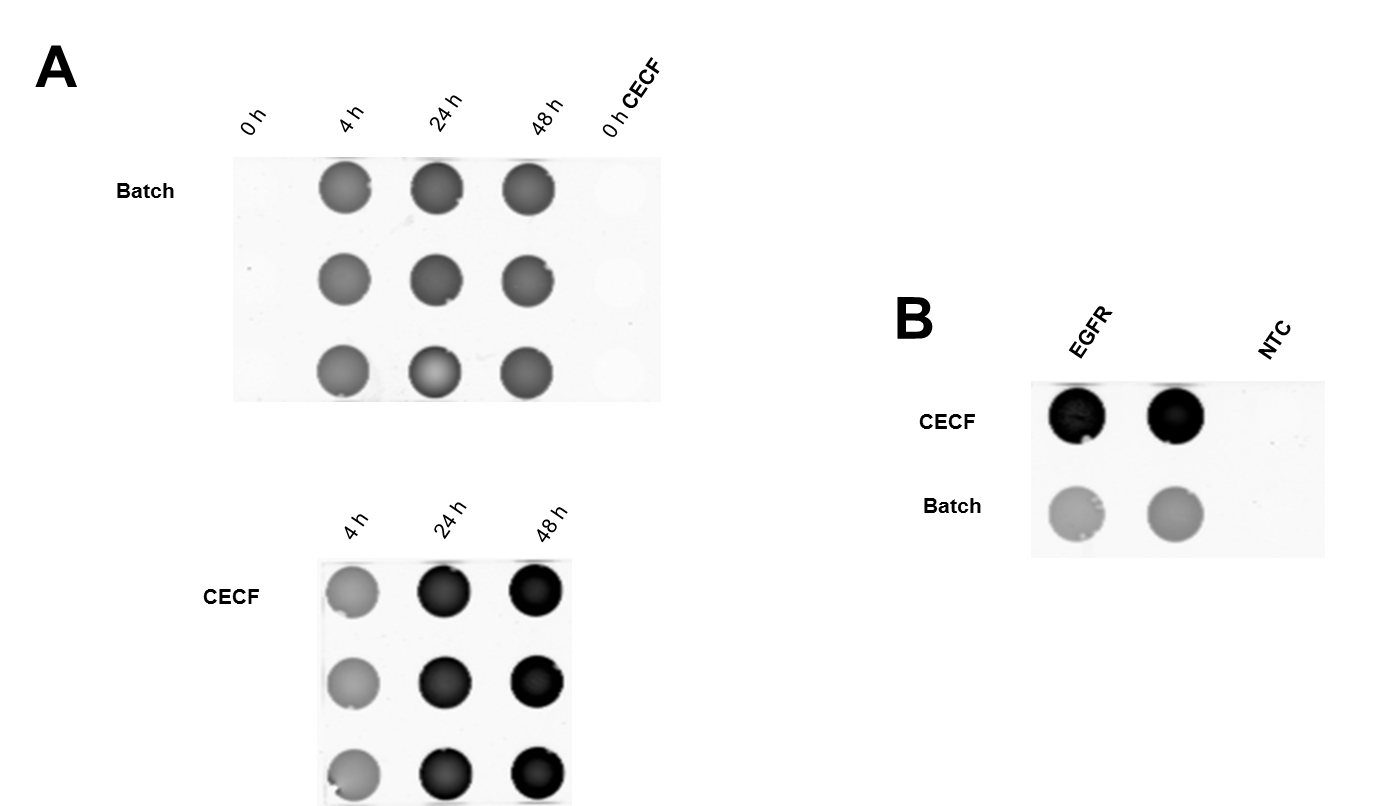


**Supplementary 7:** Original unmodified µ-Ibidi slides shown in figure 2 (A) and 3 (B) . Unspecified dotes have no relevance in this publication.


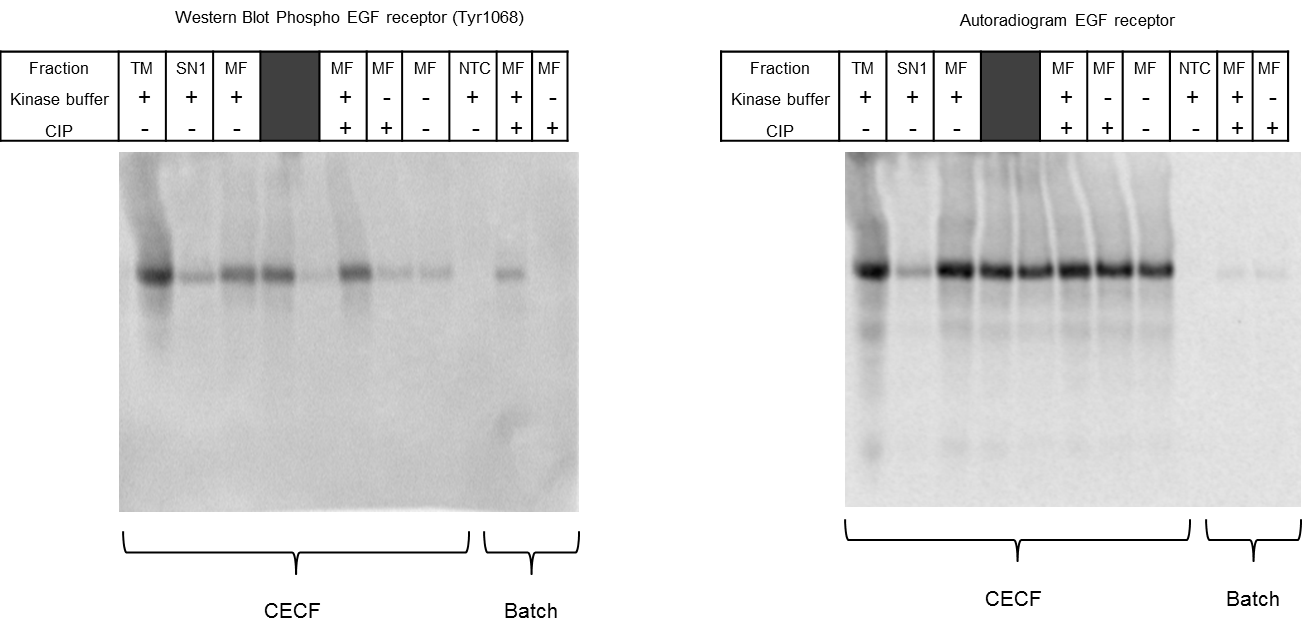


**Supplementary 8:** Original unmodified blots and corresponding autoradiograms shown in figure 3. Unspecified lanes have no relevance in this publication.


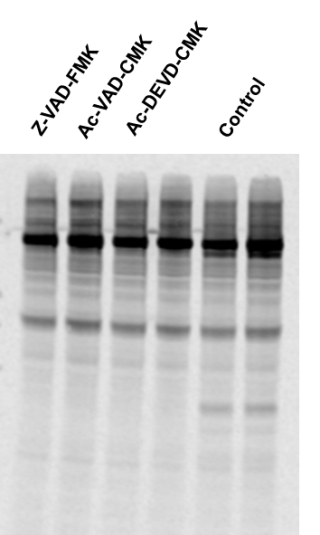


**Supplementary 9:** Original unmodified autoradiograms shown in figure 4. Unspecified lanes have no relevance in this publication.
